# Aporphine Alkaloid *N*-Nornuciferine from *Nelumbo nucifera* Induces MAPK-Associated Autophagy Under Nutrient Deprivation in Human Pancreatic Cancer Cells

**DOI:** 10.3390/ph19091454

**Published:** 2026-09-14

**Authors:** Hung Hong Nguyen, Juthamart Maneenet, Ashraf M. Omar, Tomoya Mori, Supawadee Daodee, Orawan Monthakantirat, Chantana Boonyarat, Charinya Khamphukdee, Yaowared Sumanont, Tsutomu Fujii, Suresh Awale

**Affiliations:** 1 Natural Drug Discovery Laboratory, Institute of Natural Medicine, University of Toyama, 2630 Sugitani, Toyama 930-0194, Japan; drhung103@gmail.com (H.H.N.); ashraf.omar88@gmail.com (A.M.O.); tomoya.meito@gmail.com (T.M.); 2 Department of Surgery and Science, Graduate School of Medicine and Pharmaceutical Sciences, University of Toyama, 2630 Sugitani, Toyama 930-0194, Japan; fjt@med.u-toyama.ac.jp; 3 Division of Pharmaceutical Chemistry, Faculty of Pharmaceutical Sciences, Khon Kaen University, Khon Kaen 40002, Thailand; csupawad@kku.ac.th (S.D.); oramon@kku.ac.th (O.M.); chaboo@kku.ac.th (C.B.); yaosum@kku.ac.th (Y.S.); 4 Division of Pharmacognosy and Toxicology, Faculty of Pharmaceutical Sciences, Khon Kaen University, Khon Kaen 40002, Thailand; charkh@kku.ac.th

**Keywords:** *Nelumbo nucifera*, (−)-*N*-nornuciferine, anti-austerity, pancreatic cancer, MAPK signaling, autophagy

## Abstract

**Background:** Pancreatic cancer cells adapt to nutrient stress by activating metabolic pathways that facilitate survival and proliferation within the tumor microenvironment, a phenomenon known as austerity. Targeting this resilience presents a strategy for developing chemotherapeutic agents with anti-austere properties. **Methods:** Phytochemical investigation of *Nelumbo nucifera* petals led to the isolation of six alkaloids: three benzylisoquinolines (**1**–**3**), two aporphines (**4**, **5**), and one proaporphine (**6**). Preferential cytotoxicity under nutrient deprivation was evaluated. The most active compound was further analyzed in MIA PaCa-2 cells using proliferation, migration, colony formation, and three-dimensional spheroid growth assays, with additional selective cytotoxicity assessment in KLM-1 cells. Mechanistic studies examined MAPK family signaling, autophagy-associated alterations, and apoptosis. **Results:** (−)-*N*-nornuciferine (**5**) exhibited considerable preferential cytotoxicity against MIA PaCa-2 cells (PC_50_ = 0.53 µM) and KLM-1 cells (PC_50_ = 1.11 µM). Under nutrient-rich conditions, **5** suppressed the proliferation, migration, colony formation, and spheroid growth of MIA PaCa-2 cells. Under nutrient deprivation, **5** increased MAPK phosphorylation and LC3-II accumulation while reducing p62/SQSTM1 levels. Chloroquine (CQ) further increased LC3-II accumulation, particularly at 10 µM **5**, compared with CQ alone. However, autophagy inhibitors did not rescue **5**-induced loss of viability, and the pan-caspase inhibitor Z-VAD-FMK also failed to significantly mitigate cytotoxicity. No detectable PARP or caspase-3 cleavage was observed. **Conclusions:** (−)-*N*-nornuciferine (**5**) exhibits anti-austere and anticancer activities in pancreatic ductal adenocarcinoma (PDAC) models, accompanied by MAPK phosphorylation and autophagy-related changes under nutrient deprivation conditions. Conventional caspase-dependent apoptosis is unlikely to be the main contributor to this process.

## 1. Introduction

Pancreatic cancer imposes a substantial global health and economic burden and is one of the leading causes of cancer-related mortality worldwide [1]. Its poor prognosis is largely attributable to late-stage diagnosis arising from nonspecific early symptoms, resulting in a five-year survival rate below 10% [2]. Moreover, the global incidence of pancreatic cancer is projected to increase markedly in the coming decades, potentially reaching one million new cases by 2050 [3]. Although local curative therapies, such as surgical resection and radiotherapy, are available, they are often not feasible because of the deep anatomical location of the pancreas and the high proportion of patients presenting with metastatic disease at diagnosis [4]. Consequently, gemcitabine (GEM) and its combination regimens remain the standard first-line chemotherapy for locally advanced or metastatic pancreatic cancer [5]. However, their clinical efficacy is severely limited by conventional chemoresistance [6], and GEM exhibits reduced cytotoxicity under nutrient-deprived medium (NDM) conditions, which resemble the poorly vascularized cores of pancreatic tumors [7]. GEM is typically administered as monotherapy in patients with poor performance status. Nevertheless, its curative effectiveness is seldom attained and is often linked to high recurrence rates [8]. Optimized clinical outcomes are achieved with combination therapies such as FOLFIRINOX, GEM/cisplatin, and GEM/albumin-bound paclitaxel. However, these regimens frequently face resistance and can cause severe side effects, resulting in the cessation of treatment [9]. The limitations of existing chemotherapeutic agents highlight the necessity of developing novel therapeutic approaches to combat chemoresistance and enhance treatment efficacy, particularly within the complex and nutrient-deficient pancreatic tumor microenvironment (TME). Hypovascularity is a fundamental characteristic of the pancreatic tumor microenvironment (TME), leading to persistent nutrient deprivation and posing substantial challenges for effective therapy [10]. In response to this metabolic stress, pancreatic cancer cells adopt survival strategies collectively referred to as “austerity” [11,12]. Targeting this austerity phenotype requires approaches that integrate metabolic targeting, modulation of the tumor microenvironment, and innovative drug design.

In our previous study, an ethanolic extract of *Nelumbo nucifera* petals exhibited preferential cytotoxicity against HeLa cells (PC_50_ = 10.4 µg/mL, the concentration required to kill 50% of pancreatic cancer cells under NDM), leading to the isolation of nine benzylisoquinoline alkaloids with potent anti-austerity activity. Among these, lirinidine was found to induce rapid cell death under NDM conditions by inhibiting the Akt/mTOR pathway and promoting apoptosis via caspase-3 activation and Bcl-2 suppression [13]. Other studies have shown that *N. nucifera*-derived alkaloids can enhance the therapeutic efficacy of pancreatic cancer therapies, for example, by sensitizing pancreatic cancer cells to GEM through AMPK-mediated HMGCR downregulation and YAP inhibition [14]. Building on these findings, we investigated six additional alkaloids isolated from *N. nucifera* petals, which collectively displayed preferential cytotoxicity against the MIA PaCa-2 human pancreatic cancer cell line under NDM, with a PC_50_ value of 14.4 µg/mL, and without detectable toxicity in nutrient-rich Dulbecco’s Modified Eagle Medium (DMEM). Among the isolated alkaloids, (−)-*N*-nornuciferine (**5**) emerged as a highly potent compound (PC_50_ = 0.53 µM), prompting us to elucidate the molecular mechanism underlying its anti-pancreatic cancer effects.

## 2. Results

### 2.1. Phytochemical Investigation of Nelumbo Nucifera

**Figure 1:**
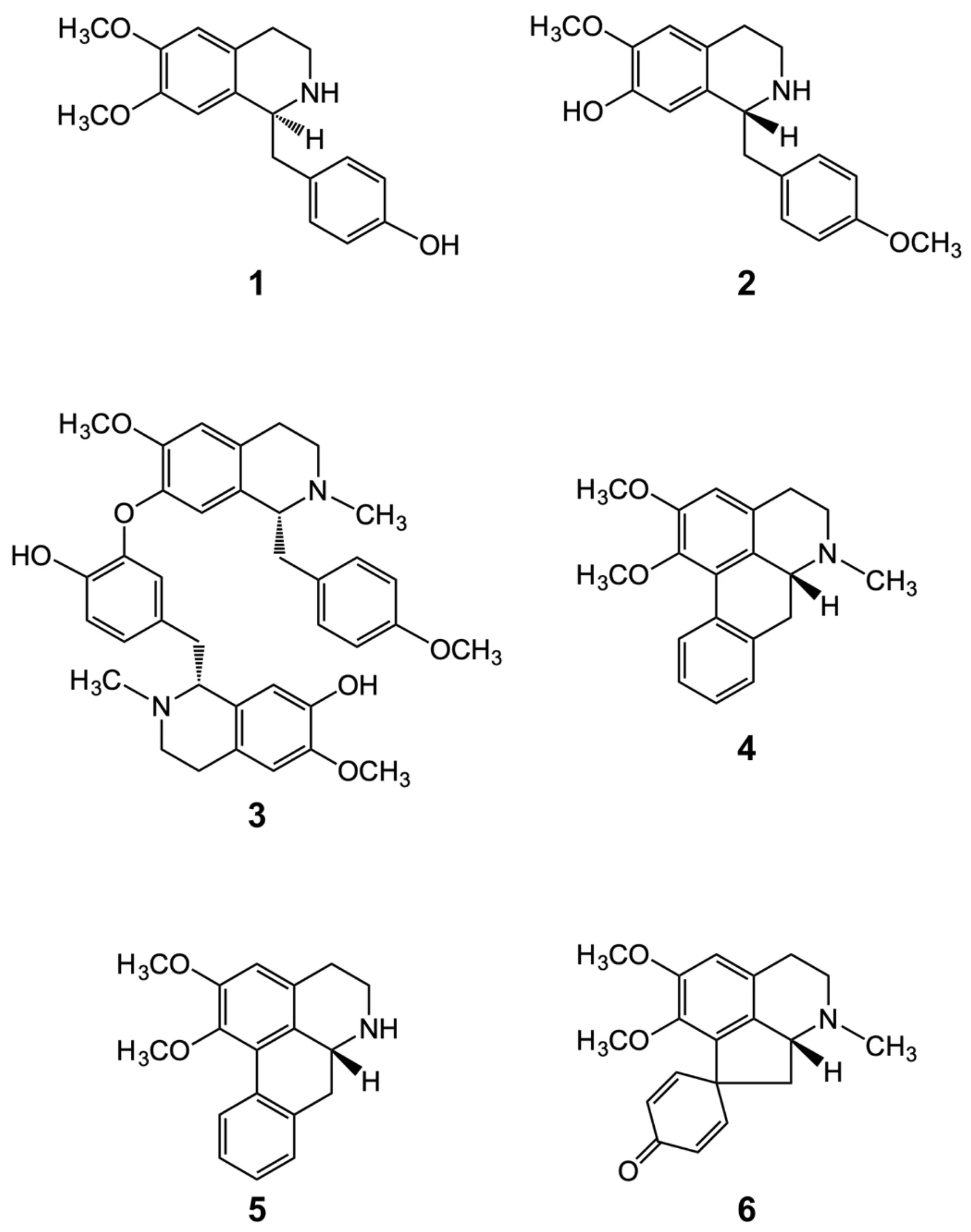
Chemical structures of benzylisoquinoline alkaloids (**1**–**3**), aporphine alkaloids (**4**, **5**), and proaporphine alkaloid (**6**) isolated from *Nelumbo nucifera* petals.

The ethanolic extract of *N. nucifera* petals was partitioned into ethyl acetate (EtOAc) and water-soluble fractions. The water-soluble fraction was purified using chromatographic methods, yielding six known alkaloids that were identified by spectroscopic analysis as benzylisoquinolines: (−)-norarmepavine (**1**) [15,16], (−)-4′-*O*-methylcoclaurine (**2**) [16,17], and (−)-isoliensinine (**3**) [18]; aporphine alkaloids: (−)-nuciferine (**4**) [19] and (−)-*N*-nornuciferine (**5**) [19]; and a proaporphine alkaloid: (+)-pronuciferine (**6**) [20] (Figure 1).

### 2.2. Preferential Cytotoxicity Induced by N-Nornuciferine (**5**) Under Nutrient Deprivation

Pancreatic cancer cells, represented by the MIA PaCa-2 cell line, exhibit a remarkable capacity to survive and proliferate in nutrient-deprived environments, reflecting an austere phenotype [21]. In this study, we evaluated the cytotoxic effects of alkaloids isolated from *N. nucifera* petals on MIA PaCa-2 cells cultured in both NDM and DMEM for 24 h.

Among the tested compounds, nuciferine (**4**) and *N*-nornuciferine (**5**) exhibited the most pronounced preferential cytotoxicity, with PC_50_ values of 0.11 µM and 0.53 µM, respectively. These PC_50_ values were comparable to those of arctigenin, a well-characterized anti-austerity agent used as a positive control (Table 1). In addition, nuciferine (**4**) and *N*-nornuciferine (**5**) also showed superior selectivity indices (SI), with values of 290 and 245.3, respectively. Although nuciferine (**4**) exhibited greater potency than *N*-nornuciferine (**5**) in the preferential cytotoxicity assay, the selection of compound **5** for subsequent experiments was not solely based on the relative PC_50_ values. Nuciferine has previously been reported to enhance the sensitivity of pancreatic cancer cells to gemcitabine through AMPK-associated HMGCR downregulation and YAP inhibition [14]. In contrast, the preferential cytotoxicity of *N*-nornuciferine under nutrient deprivation and its associated cellular stress-response profile in pancreatic ductal adenocarcinoma cells have not been characterized. Therefore, compound **5** was selected as an underexplored active aporphine alkaloid for further biological and mechanistic investigations, rather than being considered more potent or superior to nuciferine.

Structurally, compounds **4** and **5** are closely related aporphine alkaloids, and *N*-nornuciferine (**5**) is an *N*-demethylated analog of nuciferine (**4**). *N*-demethylation may affect the physicochemical properties, cellular uptake, metabolism, target engagement, and/or downstream signaling responses. However, the present experiments were not designed to establish a structure–activity relationship or to determine whether *N*-demethylation directly accounts for the activity profile of compound **5**. A direct side-by-side comparison of compounds **4** and **5** in matched functional and mechanistic assays is necessary to clarify the biological consequences of this structural modification in future studies.

**Figure 2:**
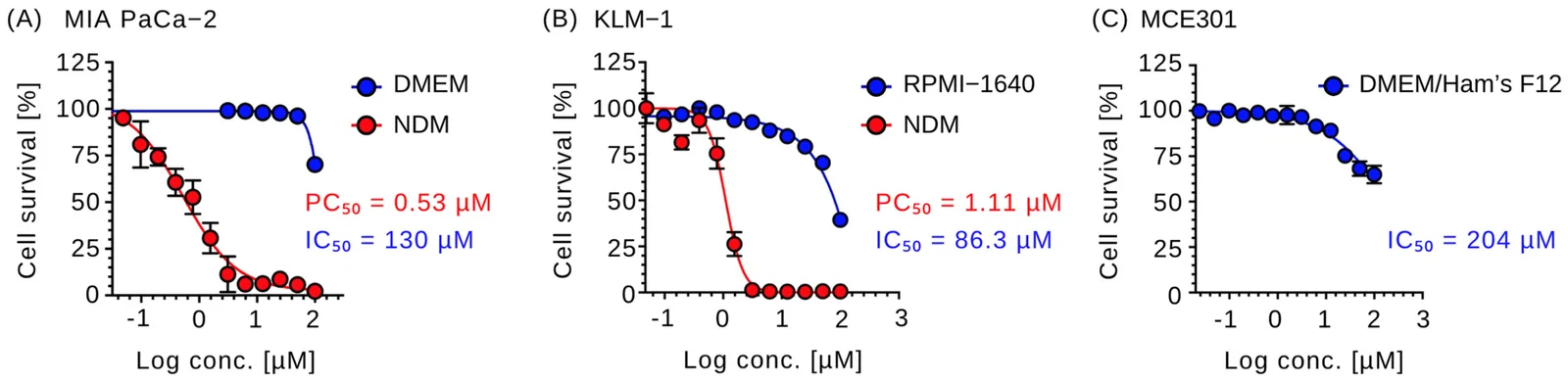
Preferential cytotoxicity of *N*-nornuciferine (**5**) against (**A**) MIA PaCa-2 cells cultured in nutrient-deprived medium (NDM, red), Dulbecco’s Modified Eagle Medium (DMEM, blue), and (**B**) another pancreatic cancer cell line, KLM-1, under NDM and RPMI-1640. (**C**) Less cytotoxicity in non-malignant immortalized colonic epithelial MCE301 cells. Data are presented as mean ± SD (*n* = 3).

Notably, **5** showed marked cytotoxicity under NDM conditions but no appreciable cytotoxicity in DMEM, with an IC_50_ value of 130 µM. In the immortalized colonic epithelial cell line MCE301, **5** displayed even lower cytotoxicity, with a higher IC_50_ value (204 µM; Figure 2C). MCE301 cells served as a non-malignant cellular comparator to assess the relative cytotoxicity of *N*-nornuciferine (**5**) towards benign cells under culture conditions. Compared to other known anti-austerity agents, such as dehydroabietinol (PC_50_ = 6.8 µM) [22] and Nic-C (PC_50_ = 2.9 µM) [23], **5** exhibited greater preferential cytotoxic potency in MIA PaCa-2 cells under NDM.

The selective cytotoxic effects of *N*-nornuciferine (**5**) were also evaluated in another pancreatic cancer cell line, KLM-1. As shown in Figure 2B, *N*-nornuciferine (**5**) killed KLM-1 cells in a concentration-dependent manner, with a PC_50_ of 1.11 µM under nutrient-deprived conditions and an IC_50_ of 86.3 µM under normal nutrient conditions (SI = 77.7). Although the potency and selectivity index differed from those observed in MIA PaCa-2 cells, these results provide preliminary evidence that **5** exerts preferential cytotoxicity under nutrient deprivation in more than one PDAC cell model.

Collectively, these findings indicate that *N*-nornuciferine (**5**) is a potent, preferentially cytotoxic alkaloid under nutrient-deprived conditions in MIA PaCa-2 and KLM-1 PDAC cells. The present results support the selection of this compound as an exploratory lead for further biological investigations. They did not establish that compound **5** is superior to nuciferine (**4**), nor did they define the structure–activity relationship between the two aporphine alkaloids.

### 2.3. Morphological Changes Analysis of MIA PaCa-2 Under Nutrient-Deprived Medium Induced by N-Nornuciferine (**5**)

Investigating cell morphology following compound exposure can help elucidate the relationship between molecular profiling and other analytical techniques, thereby providing insights into the effects of agents on cellular functions [24]. To evaluate the effect of **5** on MIA PaCa-2 pancreatic cancer cells under nutrient-deprived conditions, live-cell imaging was employed to monitor morphological changes in real-time over 24 h (Supplementary Movie S1). At the end of the experiment, a dual-fluorescence staining assay using ethidium bromide (EB) and acridine orange (AO) was performed to assess cell viability. In this assay, viable cells fluoresce green due to AO uptake, whereas EB stains dead or dying cells red, indicating compromised membrane integrity.

**Figure 3:**
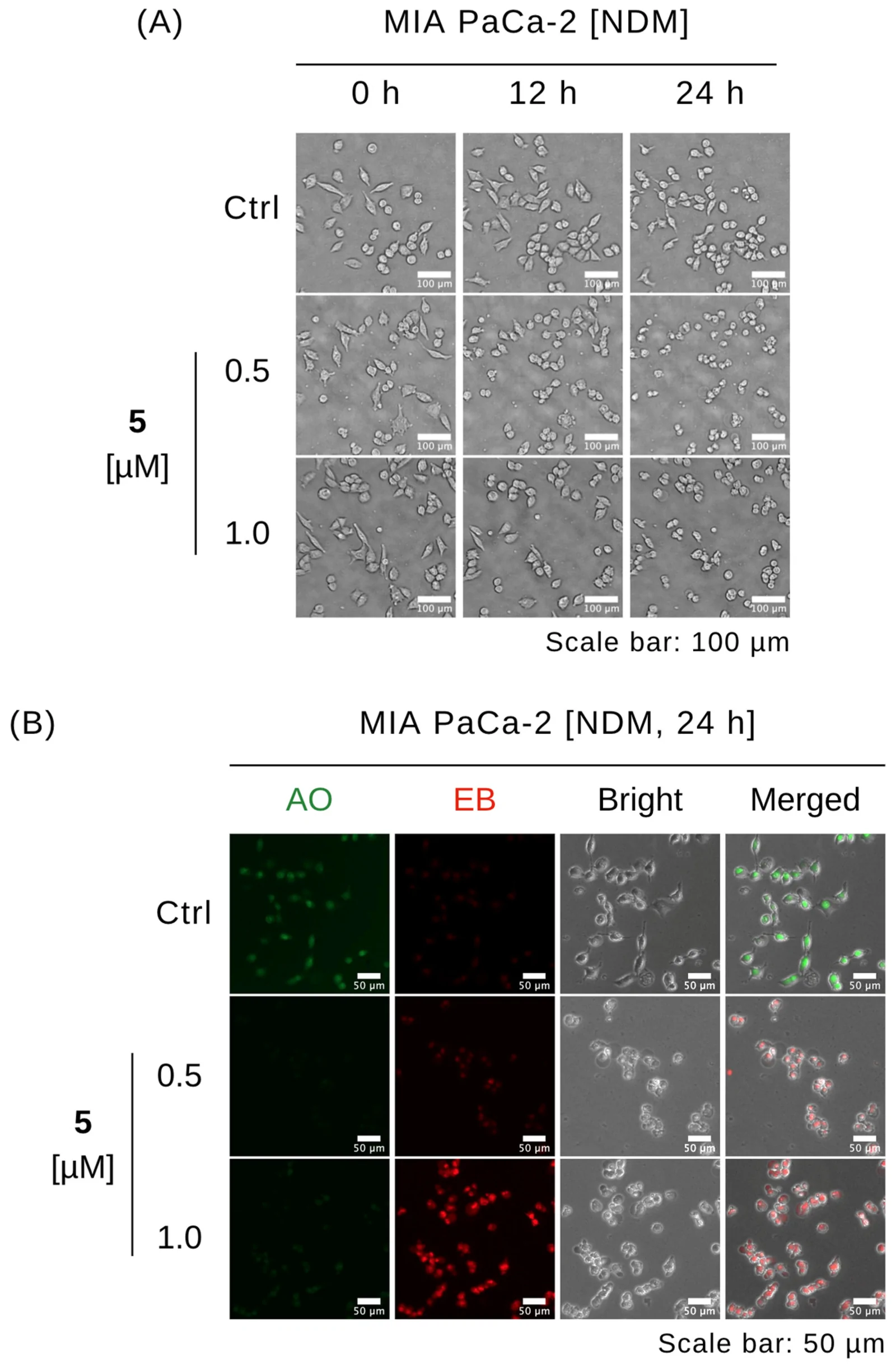
Effect of *N*-nornuciferine (**5**: 0.5 and 1 µM) on MIA PaCa-2 human pancreatic cancer cells under NDM. (**A**) Representative time-lapse images of MIA PaCa-2 cells cultured in NDM, either untreated or treated with **5**, captured at 0, 12, and 24 h using a CytoSMART digital microscope. (**B**) Morphological changes after 24 h of treatment were assessed using ethidium bromide/acridine orange (EB/AO) dual staining and visualized using an EVOS FL digital fluorescence microscope.

MIA PaCa-2 cells were treated with 0.5 and 1.0 µM concentrations of **5**, alongside an untreated control group, and incubated in NDM equipped with a CytoSMART digital microscope for 24 h. As shown in Figure 3A, untreated MIA PaCa-2 cells maintained intact morphology and normal cellular boundaries throughout the incubation period. In contrast, cells exposed to 0.5 µM of **5** exhibited noticeable shrinkage after 12 h, followed by membrane blebbing and the release of intracellular contents after 24 h. At 1.0 µM of **5**, cellular changes occurred more rapidly, with significant alterations observable within 3 h, resulting in cell death within 24 h of exposure to **5**. To confirm these observations, EB/AO staining consistently revealed uniform green fluorescence in the control cells, indicating high viability. In contrast, cells exposed to 0.5 and 1 µM compound **5** emitted predominantly red fluorescence, suggesting an inhibitory effect on cell viability and compromised membrane integrity (Figure 3B). These findings suggest the anti-austerity properties of compound **5** and reaffirm its ability to act as a selective cytotoxic agent to inhibit pancreatic cancer cells under metabolic stress.

### 2.4. Inhibition of MIA PaCa-2 Cell Proliferation in DMEM Caused by N-Nornuciferine (**5**)

Cell proliferation is a key indicator of tumor progression and drug responsiveness in cancer research [25]. In the context of anticancer drug development, the evaluation of cell proliferation provides critical insights into the capacity of candidate compounds to inhibit uncontrolled cell growth, a hallmark of malignancy, through mechanisms such as cell cycle arrest and induction of cell death [26].

**Figure 4:**
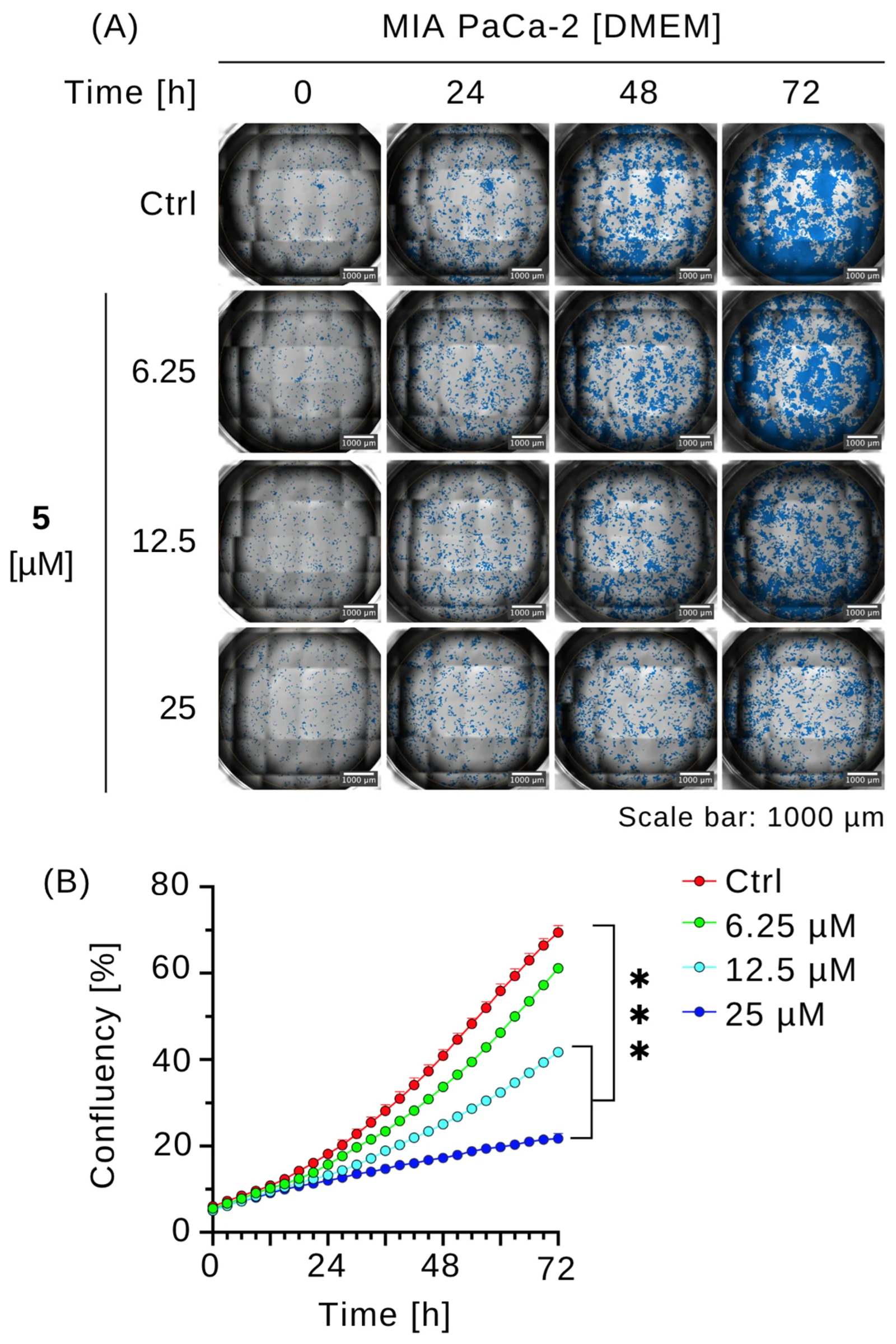
Effect of *N*-nornuciferine (**5**: 6.25, 12.5, and 25 µM) on MIA PaCa-2 cell proliferation in DMEM. (**A**) Representative brightfield images captured using the OMNI device at 0, 24, 48, and 72 h after treatment. (**B**) Quantification of MIA PaCa-2 cell confluency was performed by measuring the percentage of confluency at 3-h intervals over a 72-h period (see Supplementary Movie S2). Statistical analyses were conducted using two-way ANOVA, followed by Dunnett’s multiple comparison test (GraphPad Prism ver. 10), with *n* = 3. Significant differences compared to the control are indicated (*** *p* < 0.001).

In this study, we employed a real-time monitoring approach using an OMNI device to assess the anti-proliferative activity of **5** in MIA PaCa-2 cells (Figure 4A). MIA PaCa-2 cells were seeded in a 96-well plate and observed in DMEM. Images were captured every 3 h, resulting in 25 images being captured over 72 h. In the control group, the cells proliferated steadily, reaching approximately 70% confluence by the end of the experiment. In contrast, treatment with **5** at concentrations of 12.5 and 25 µM resulted in a marked concentration-dependent suppression of cell proliferation, reducing the confluency by approximately 40% and 20%, respectively (Figure 4B). These findings suggest that **5** exhibits significant anti-proliferative activity in DMEM, further supporting its potential as a therapeutic candidate.

### 2.5. Suppression of MIA PaCa-2 Cell Migration by N-Nornuciferine (**5**) Under Nutrient-Rich Conditions in Real-Time Monitoring

Metastasis plays a pivotal role in cancer progression and mortality, encompassing a complex sequence of events, such as angiogenesis, cellular detachment, invasion into the vasculature, and colonization of distant, nutrient-rich organs [27,28]. Consequently, the inhibition of cancer cell migration under normal nutrient conditions is critical for the development of anti-metastatic therapies [27]. To evaluate the antimigratory potential of **5**, we performed a real-time wound-healing assay using a CytoSMART digital microscopy system to monitor the migration of MIA PaCa-2 pancreatic cancer cells in DMEM.

**Figure 5:**
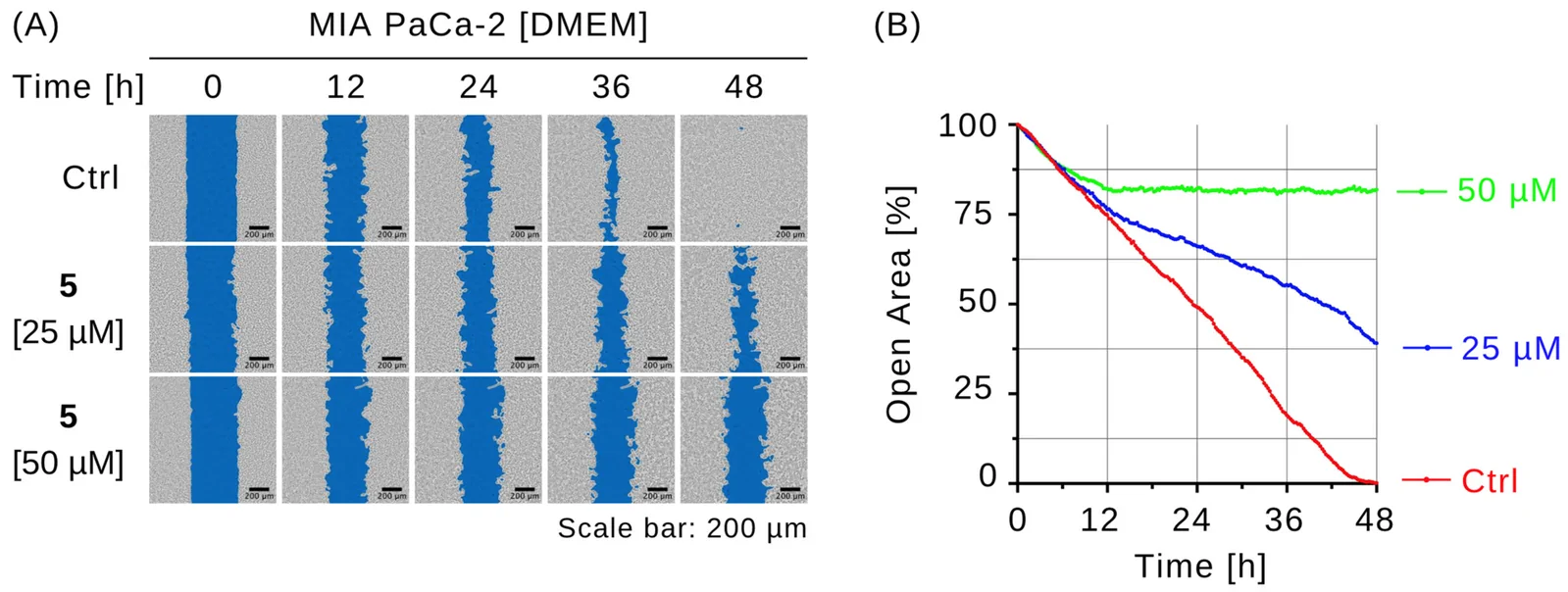
Inhibitory effect of *N*-nornuciferine (**5**: 25 and 50 µM) on the migration of MIA PaCa-2 human pancreatic cancer cells under DMEM conditions. (**A**) Real-time bright-field images of the wound area (blue area) captured at 15-min intervals over 48 h using the CytoSMART digital microscopy system (Supplementary Movie S3). Representative images of cell-free gap closure at 0, 12, 24, 36, and 48 h in untreated cells and cells treated with **5**. (**B**) Quantification of the open area (as a percentage of the initial gap) was performed at each time point using bright-field imaging analysis.

MIA PaCa-2 cells were seeded in a 2-well culture insert and incubated for 24 h at 37 °C in a 5% CO_2_ incubator. Subsequently, the insert was removed to create an approximately 500-μm cell-free gap. The culture medium of the treated groups was replaced with fresh DMEM containing 25 and 50 µM **5**, whereas the untreated group received only DMEM. Time-lapse bright-field images were captured every 15 min for 48 h, generating 193 frames (Figure 5A, Supplementary Movie S3).

Untreated control MIA PaCa-2 cells rapidly filled the gap, reducing the open area by approximately 50% at 24 h and completely closing the gap within 48 h. In contrast, cells treated with **5** at 25 and 50 µM exhibited significantly impaired migration, with the wound area remaining approximately 37% and 81% open at 48 h, respectively (Figure 5B). These real-time observations demonstrated that **5** markedly suppressed directional cell movement and prevented effective scratch closure. Collectively, these findings suggest that **5** disrupts the migratory behavior of pancreatic cancer cells under nutrient-rich conditions, indicating its potential as an anti-metastatic agent for the treatment of pancreatic cancer.

### 2.6. Inhibition of MIA PaCa-2 Colony Formation by N-Nornuciferine (**5**) Under Nutrient-Rich Conditions

The establishment of secondary colonies in nutrient-rich distant organs characterizes metastatic progression of pancreatic cancer. Consequently, the prevention of colony formation is regarded as a pivotal strategy for developing anti-metastatic therapies [25]. Several anti-austerity agents, such as grandifloridin D [29] and calliviminone A [30], have been reported to inhibit colony formation in in vitro models of human pancreatic cancer cells. In this study, we investigated the clonogenic inhibitory potential of **5** under nutrient-rich conditions using MIA PaCa-2 cells.

To assess the anti-clonogenic ability of compound **5**, MIA PaCa-2 cells were seeded into 24-well plates and allowed to adhere overnight. The following day, the cells were exposed to varying concentrations of **5** (0, 1.25, 2.5, 5, 10, and 20 µM) for 24 h in DMEM. Once the treatment was concluded, the medium was exchanged for fresh DMEM, and colonies were observed over a period of 10 d. The area occupied by the colonies was measured relative to the total surface area of each well.

**Figure 6:**
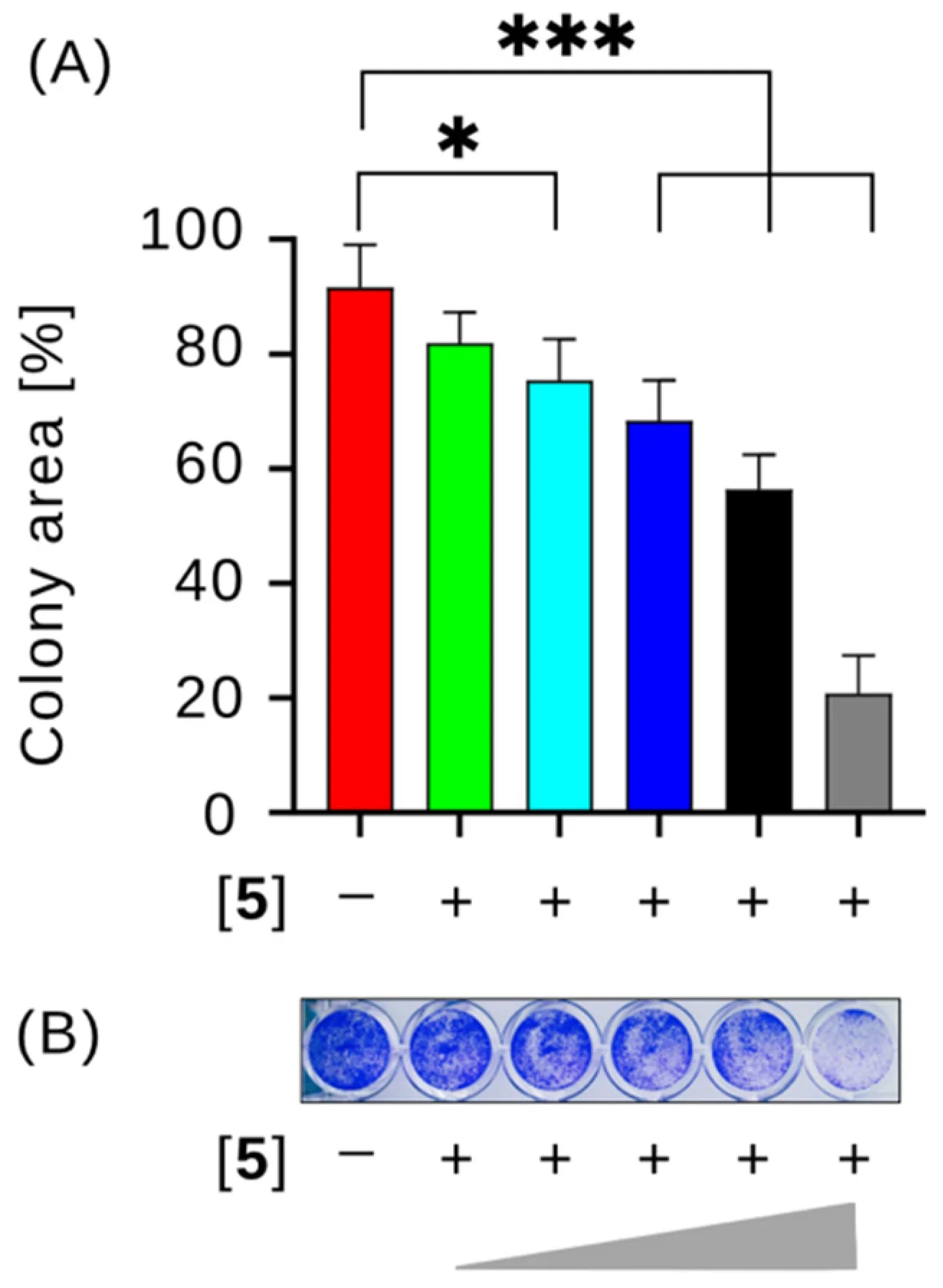
Inhibitory effect of *N*-nornuciferine (**5**: 1.25, 2.5, 5, 10, and 20 µM) on MIA PaCa-2 colony formation in nutrient-rich DMEM. (**A**) Quantification of colony area percentage following 24-h treatment with *N*-nornuciferine (**5**) and a 10-day incubation period. Data are presented as mean ± SD (*n* = 4). * *p* < 0.05 and *** *p* < 0.001 vs. control (one-way ANOVA with Dunnett’s multiple comparisons test). (**B**) Representative images of colonies at the indicated concentrations of **5**.

In the untreated control group, MIA PaCa-2 cells formed large colonies that occupied approximately 90% of the well area. In contrast, **5** significantly suppressed colony formation in a concentration-dependent manner. At 2.5 µM (cyan column), the colony area was reduced to approximately 77% (* *p* < 0.05), whereas at 20 µM (gray column), it decreased to approximately 20% (*** *p* < 0.001 vs. control), indicating a substantial reduction in colony number and size following staining (Figure 6). These findings demonstrate that compound **5** effectively inhibits colony formation in DMEM, suggesting its potential to disrupt key metastatic processes, such as cell adhesion, proliferation, survival, and establishment of secondary tumors in distant organs.

### 2.7. Inhibition of Spheroid Growth by N-Nornuciferine (**5**) in the Real-Time 3D Spheroid Culture Model

**Figure 7:**
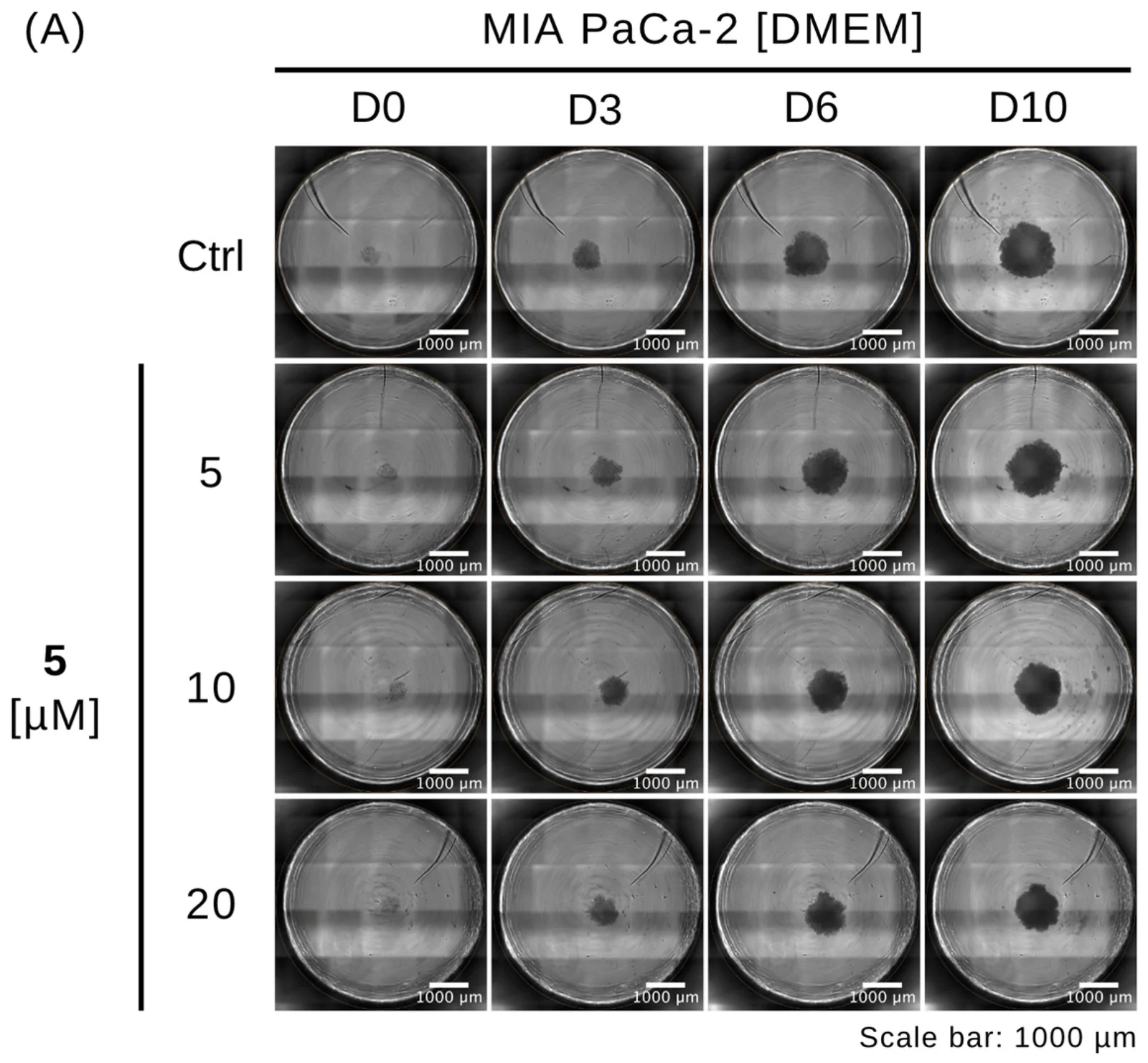

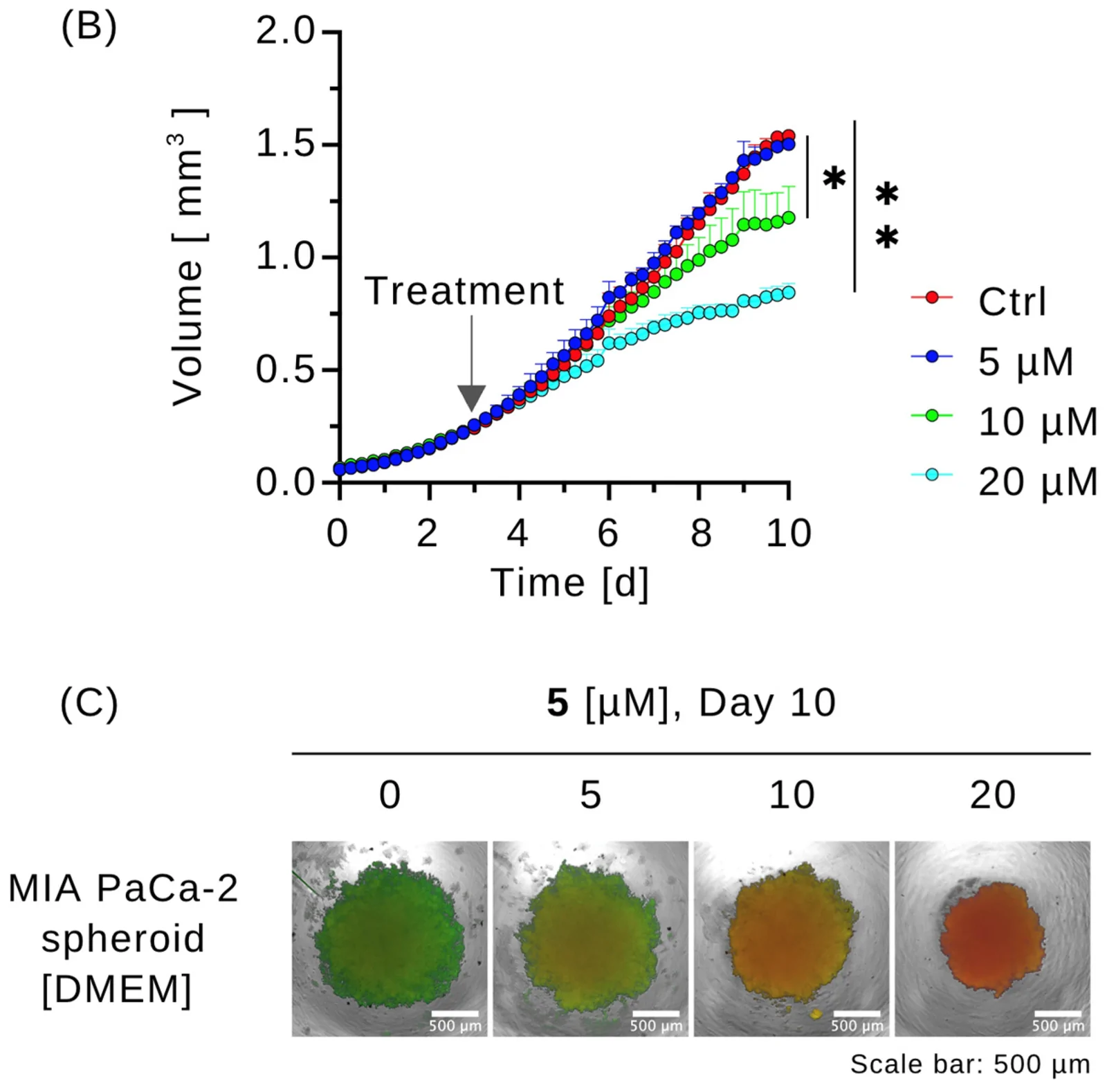
Inhibitory effect of *N*-nornuciferine (**5**: 5, 10, and 20 µM) on MIA PaCa-2 spheroid formation. (**A**) Representative images of MIA PaCa-2 spheroids cultured in DMEM (control) or treated with **5**, captured by the OMNI device on days 0, 3, 6, and 10 (scale bar = 1000 µm). (**B**) Quantitative analysis of spheroid volume over 10 d, measured at 6-h intervals (*n* = 3). Data are presented as mean ± SEM. * *p* < 0.05 and ** *p* < 0.01 vs. control (two-way ANOVA with Dunnett’s multiple comparison test). (**C**) Fluorescence images of spheroids stained with ethidium bromide/acridine orange (EB/AO) on day 10, indicating their viability. Green fluorescence denotes viable cells, whereas red fluorescence indicates non-viable cells. Scale bar = 500 µm.

Conventional two-dimensional (2D) cell culture systems fail to adequately replicate the complexity of the TME, particularly in PDAC, where hypoxia and nutrient deprivation contribute to resistance to conventional therapy [21,31,32]. To better mimic the complexity of the pancreatic TME, we employed a three-dimensional (3D) spheroid culture model to assess the effect of **5** on MIA PaCa-2 spheroid growth. This 3D spheroid culture model facilitates cell-to-cell and cell-to-matrix interactions, promotes the formation of hypoxic cores, and simulates the drug penetration barrier characteristics of PDAC tumors [33,34,35]. Spheroid expansion and cell viability were monitored for 10 d using real-time imaging and quantitative analyses (Supplementary Movie S4). MIA PaCa-2 cell spheroids were either untreated or treated with **5** at concentrations of 5, 10, or 20 µM from day 3, after reaching an average diameter of approximately 700 µm. Before treatment initiation on day 3, spheroids were generated under identical conditions using the same cell seeding density and centrifugation procedure. The spheroid projected area and equivalent diameter were quantified from bright-field images using an automated ImageJ (version 2.16.0/1.54p) macro, and the estimated spheroid volume was calculated from the projected area assuming spherical geometry. As the volume was derived from two-dimensional images, it was presented as an estimated rather than a directly measured three-dimensional parameter. As shown in Figure 7A, spheroid formation was tracked on days 0, 3, 6, and 10 after seeding. No difference was observed between the untreated spheroids and those treated with 5 µM of **5**, and they continued to grow. However, spheroids exposed to 10 and 20 µM concentrations exhibited concentration-dependent growth inhibition, with significantly reduced spheroid volume by day 10. Quantitative analysis (Figure 7B) revealed that the control spheroids reached an average estimated volume of approximately 1.5 mm^3^ by day 10. No significant difference was observed between the control and 5 µM treatment groups. In contrast, spheroids treated with 10 and 20 µM of **5** showed reductions in estimated volume of approximately 25% (* *p* < 0.05) and 45% (** *p* < 0.01), respectively. At the end of the experiment, cell viability within the spheroids was evaluated using EB/AO dual-staining reagents (Figure 7C). The control spheroids exhibited predominantly green fluorescence, indicating intact and viable cells. In contrast, treatment with **5** resulted in a concentration-dependent increase in red fluorescence, indicating compromised cell viability. Treatment with 20 µM displayed the strongest red fluorescence, suggesting a predominance of non-viable cells. Because EB/AO imaging was used as an endpoint assessment, these fluorescence observations indicate relative changes in membrane integrity and cell viability; however, they do not independently establish a specific cell death mechanism. This observation corresponds to a reduction in the spheroid volume.

PDAC spheroids are known for their resistance to gemcitabine, which is attributed to inadequate drug penetration and enhanced survival signaling pathways under conditions mimicking the TME [36,37]. The ability of **5** to disrupt spheroid growth suggests its potential to overcome these obstacles, possibly by interfering with cell–cell interactions and inducing cellular compromise in the spheroid core. This concentration-dependent cytotoxicity is consistent with the broader understanding of how targeted therapies disrupt cancer cell survival pathways in PDAC. The efficacy of **5** in inducing cell death, as demonstrated by increased EB staining in spheroids treated with higher concentrations, indicates that it may be a promising candidate for further investigation in preclinical PDAC models.

### 2.8. N-Nornuciferine (**5**) Induces Autophagy-Associated, Caspase-Independent Changes Accompanied by MAPK Activation Under Nutrient-Deprived Conditions

The MAPK pathway is a signaling cascade that governs cellular processes, including proliferation, differentiation, survival, and apoptosis [38]. Activation begins with extracellular growth factors stimulating RAS, which triggers MEK phosphorylation and downstream modulation of transcription factors and effector proteins, thereby influencing gene expression and cell fate [39]. In PDAC, MAPK signaling is frequently hyperactivated by KRAS mutations [40]. In various cancers, including pancreatic cancer, autophagy, a lysosome-mediated degradation and recycling mechanism, is a double-edged sword [41]. Although it promotes cell survival under stress conditions, excessive autophagy can contribute to cell death [42]. Additionally, apoptosis-based drug resistance is a major contributor to therapeutic failure and disease aggressiveness in pancreatic cancer [43]. There is ample evidence indicating complex interactions among MAPK signaling, apoptosis, and autophagy in pancreatic cancer [39]. In KRAS-driven PDAC tumors, MAPK pathway activation enhances both apoptosis and autophagy, particularly under nutrient-deprived or hypoxic conditions. Therefore, the heightened activation of MAPK family pathways, which augments autophagic activity and apoptosis, may contribute to the death of pancreatic cancer cells [44].

**Figure 8:**
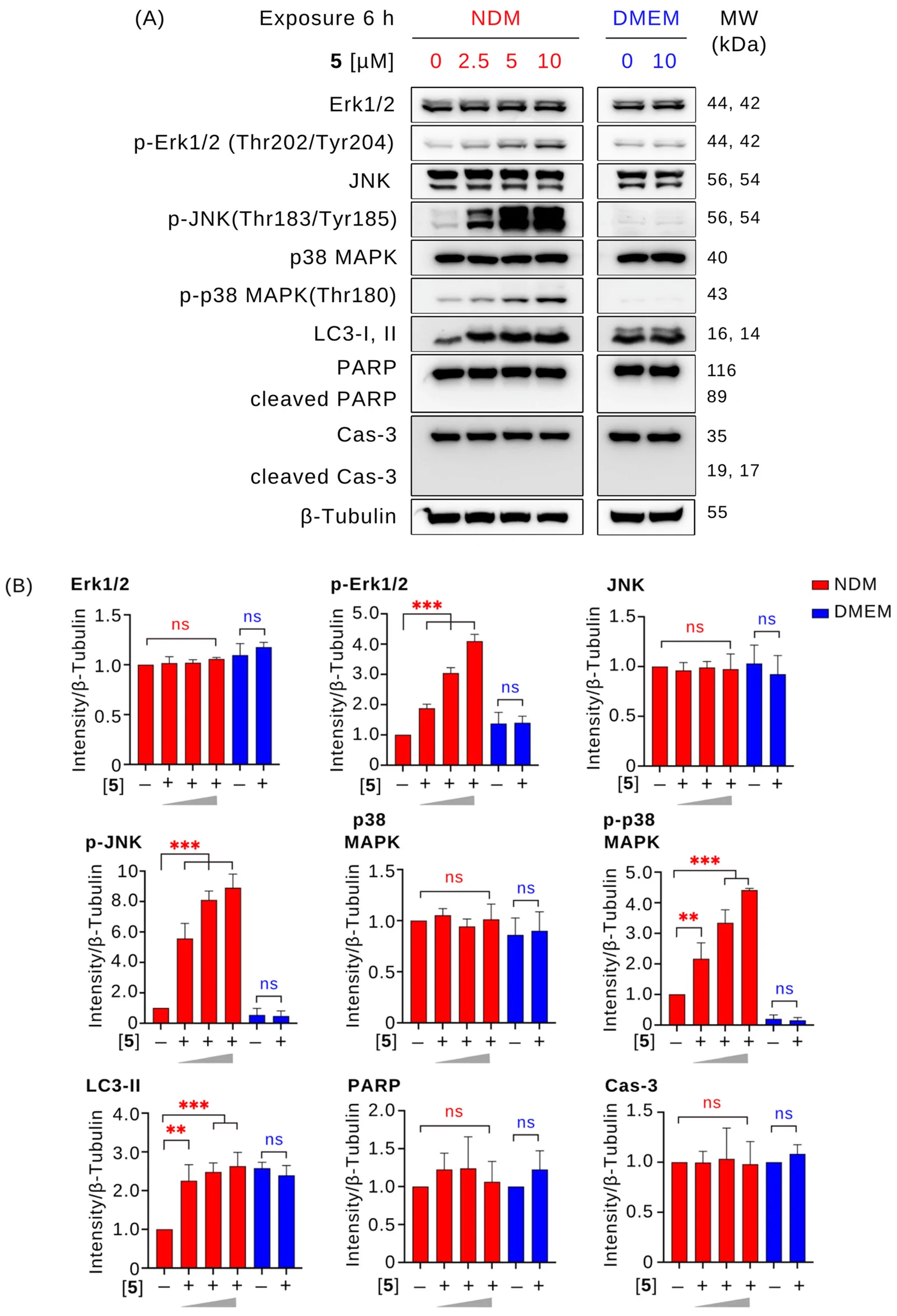
Western blot analysis of (**A**) MAPK family signaling pathways, their phosphorylation status, autophagy, and apoptosis-related proteins. MIA PaCa-2 cells were treated with **5** at concentrations of 0, 2.5, 5, and 10 µM in NDM; 0 and 10 µM in DMEM for 6 h. Blots show total and phosphorylated forms of Erk1/2, p-Erk1/2 (Thr202/Tyr204), JNK, p-JNK (Thr183/Tyr185), p38 MAPK, and p-p38 MAPK (Thr180), along with autophagy markers LC3-I and LC3-II and apoptosis-related proteins PARP and Caspase-3, including their cleaved forms. β-tubulin was used as the loading control for Western blotting. (**B**) Quantitative analysis of protein levels normalized to β-tubulin from three independent experiments. (Data are presented as mean ± SEM, *n* = 3, ns = not significant, ** *p* < 0.01, *** *p* < 0.001 vs. control).

Numerous studies have elucidated the mechanisms by which alkaloids derived from *N. nucifera* induce cancer cell death, notably through the activation of the MAPK pathway and autophagy in pancreatic cancer cells [45]. However, the specific mechanism of action of *N*-nornuciferine (**5**) in pancreatic cancer cell lines remains unclear. Therefore, to elucidate the mechanism resulting in cell death induced by **5**, we conducted Western blot analysis to evaluate the expression levels of key proteins involved in MAPK family signaling pathways, autophagy (LC3-I and LC3-II), and apoptosis (PARP and caspase-3, including their cleaved forms) under both NDM and DMEM conditions. MIA PaCa-2 cells were treated with various concentrations of compound **5** (NDM: 0, 2.5, 5, and 10 µM; DMEM: 0 and 10 µM) for 6 h. The 6 h time point was selected based on real-time morphological imaging (Figure 3), which showed that *N*-nornuciferine (**5**)-induced membrane changes and loss of viability became detectable as early as 3 h under nutrient deprivation, indicating that this time window captures an early, biologically relevant stress response preceding overt cell death. As shown in Figure 8A,B, **5** induced a concentration-dependent increase in the phosphorylation of stress-responsive MAPK family proteins (p-Erk1/2, p-JNK, and p-p38 MAPK) under nutrient-deprived conditions. The total protein levels of MAPK components remained unchanged. Interestingly, in DMEM, the phosphorylated signals were significantly weaker, even at an equivalent concentration of 10 µM of **5**. The overall expression levels of PARP and caspase-3 did not exhibit significant suppression, nor was there an increase in the levels of cleaved PARP or cleaved caspase-3. This indicates that *N*-nornuciferine (**5**) did not induce detectable activation of the canonical apoptotic markers, specifically PARP and caspase-3 cleavage, at the early time point of 6 h.

As illustrated in Figure 8A, there was a notable concentration-dependent accumulation of LC3-II in the presence of NDM, with a pronounced increase in expression observed at 2.5 µM (Figure 8B). This suggests that compound **5** influences the autophagic process. Therefore, to further elucidate the role of **5** in autophagic cell death under nutrient-deficient conditions, we assessed autophagic flux using co-treatment with chloroquine (CQ) and 3-methyladenine (3-MA). CQ functions as a late-stage autophagic inhibitor by preventing autophagosome-lysosome fusion [46], whereas 3-MA is a type III phosphatidylinositol 3-kinase (PI3K) inhibitor essential for early-stage autophagic suppression [47]. Western blotting was performed to evaluate the expression of LC3-II and p62 (SQSTM1). p62 is an autophagy substrate that is degraded inside autolysosomes and serves as a key indicator of autophagic flux. Typically, low p62 levels are indicative of active autophagy, whereas the accumulation of p62 suggests inhibition of the autophagic process [48]. In both the single treatment with **5** and the combined treatment with 3-MA, p62 levels consistently decreased, indicating modulation of autophagic flux induced by **5**.

**Figure 9:**
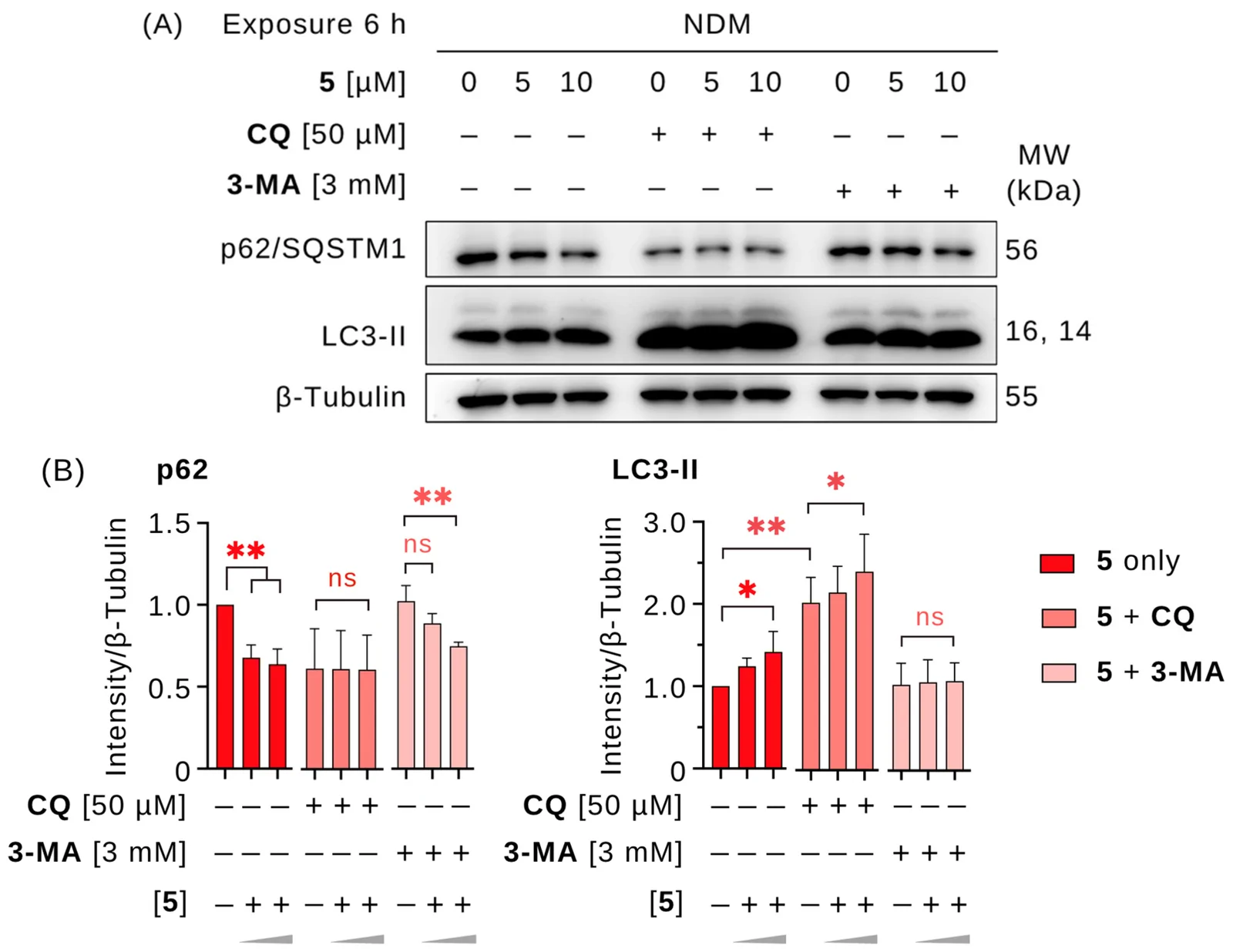
(**A**) Western blot analysis of autophagy-associated markers in cells treated with *N*-nornuciferine (**5**) alone or in combination with CQ (50 µM) or 3-MA (3 mM) under nutrient-deprived medium (NDM) conditions for 6 h. (**B**) Quantification of p62/SQSTM1 and LC3-II protein levels. *N*-nornuciferine (**5**) treatment decreased p62/SQSTM1 levels and increased LC3-II accumulation, while CQ co-treatment resulted in further LC3-II accumulation, particularly at 10 µM *N*-nornuciferine (**5**), compared to CQ alone. These findings support the induction of autophagy-associated activity by *N*-nornuciferine (**5**) under nutrient-deprived conditions. Data are presented as mean ± SD (*n* = 3). ns, not significant; * *p* < 0.05; ** *p* < 0.01.

CQ treatment alone significantly increased LC3-II accumulation under nutrient deprivation. Importantly, co-treatment with *N*-nornuciferine (**5**) at a concentration of 10 µM further elevated LC3-II levels compared to CQ treatment alone (Figure 9B, * *p* < 0.05). Meanwhile, *N*-nornuciferine (**5**) at 5 µM did not significantly affect LC3-II accumulation compared to CQ alone. This suggests that **5** promotes additional LC3-II accumulation beyond that induced by the pharmacological inhibition of lysosomal degradation. The accumulation of LC3-II was evident even in the presence of CQ, indicating substantial autophagosome release. These results are consistent with ongoing autophagic activity and underscore the role of autophagy in response to treatment with **5** (Figure 9A,B).

**Figure 10:**
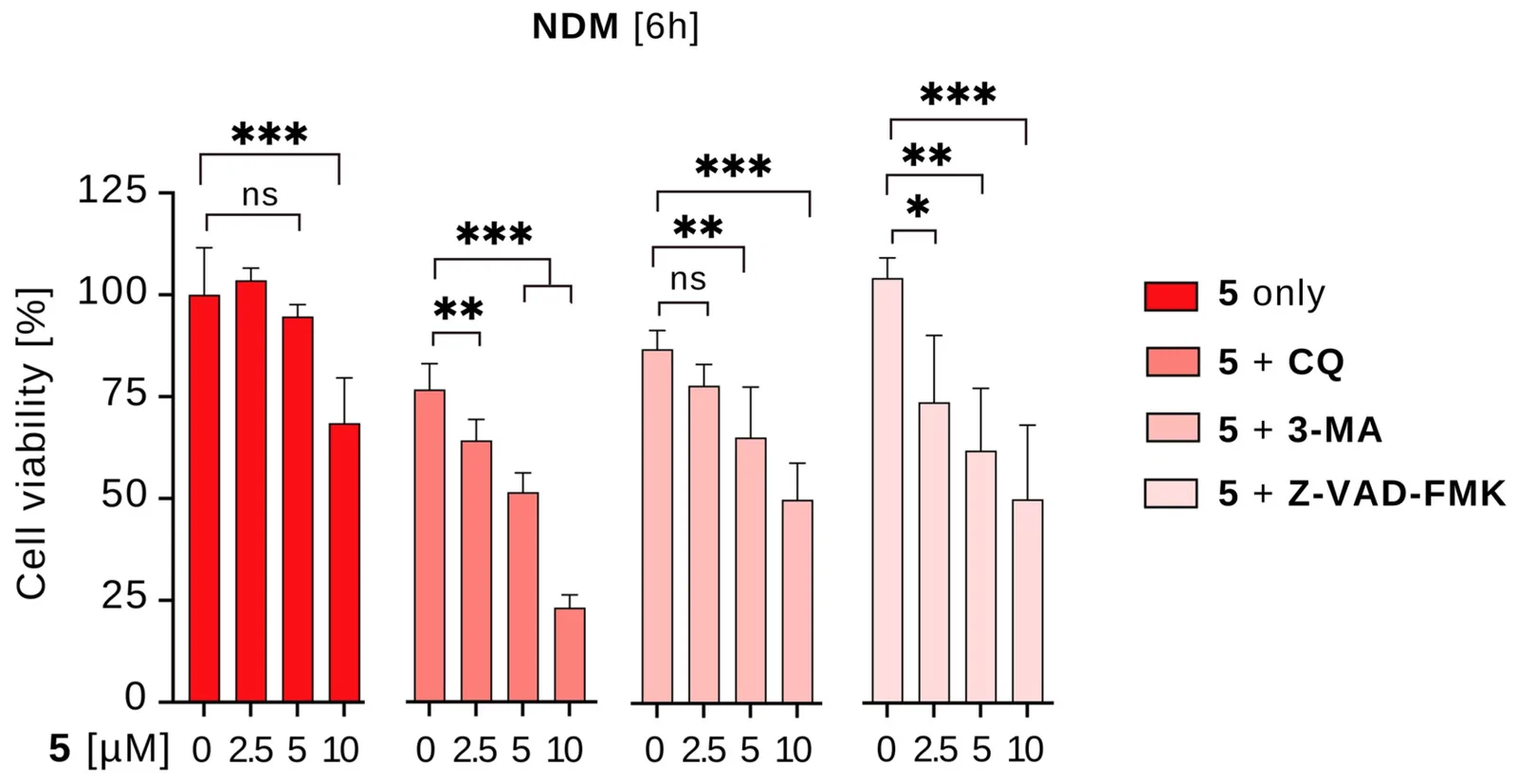
Pharmacological inhibition of autophagy and caspase activity in *N*-nornuciferine (**5**)-treated cells. Cells were pretreated with CQ (50 μM), 3-MA (3 mM), or Z-VAD-FMK (20 μM) for 1 h and subsequently treated with *N*-nornuciferine (**5**) (0, 2.5, 5, and 10 μM) under nutrient deprivation. Cell viability was determined using the CCK-8 assay after 6 h of *N*-nornuciferine (**5**) treatment and normalized to that of the untreated control. Data are expressed as mean ± SD, *n* = 3 (ns = not significant, * *p* < 0.05, ** *p* < 0.01, *** *p* < 0.001 vs. control).

To investigate the roles of autophagy and caspase-dependent apoptosis in the reduction in cell viability induced by *N*-nornuciferine (**5**), pharmacological inhibition studies were conducted under nutrient-starved conditions for 6 h. Cells were treated with *N*-nornuciferine (**5**) in the presence or absence of the autophagy inhibitors CQ or 3-MA or the pan-caspase inhibitor Z-VAD-FMK, followed by cell viability assessment using the CCK-8 assay. This study aimed to determine whether pharmacological inhibition of autophagy or caspase activity could alleviate the cytotoxic effects of *N*-nornuciferine (**5**). Figure 10 illustrates that pretreatment with pharmacological inhibitors of autophagy, specifically CQ or 3-MA, in conjunction with the pan-caspase inhibitor Z-VAD-FMK, did not significantly improve cell viability after exposure to *N*-nornuciferine (**5**). These findings suggest that autophagy is not crucial for cell death and that caspase activity does not substantially contribute to cytotoxicity under the experimental conditions used in this study.

Collectively, these observations suggest that *N*-nornuciferine (**5**) induces autophagy-related alterations accompanied by a loss of cell viability, particularly under nutrient-deprived conditions. Although CQ and 3-MA failed to rescue the viability of **5**-treated cells, the observed LC3-II accumulation and p62/SQSTM1 reduction indicated an autophagy-associated cellular response to **5** under metabolic stress. Additionally, the absence of detectable apoptotic markers, together with the lack of significant viability rescue by the pan-caspase inhibitor Z-VAD-FMK, supports a predominantly caspase-independent mode of cell death. *N*-nornuciferine (**5**) also induced the phosphorylation of multiple MAPK family proteins, which was associated with the observed autophagy-related changes. However, the causal relationship between MAPK activation, autophagy, and loss of cell viability remains to be established. Overall, these findings suggest that *N*-nornuciferine (**5**) exploits cellular responses to metabolic stress and represents a potential lead for targeting nutrient-dependent vulnerabilities in PDAC cells.

## 3. Discussion

Pancreatic cancer is a highly aggressive malignancy that exhibits significant resistance to conventional chemotherapy. This resistance is primarily due to the ability of cancer cells to undergo metabolic adaptation in response to nutrient deficiencies within the tumor microenvironment, a phenomenon known as austerity. This adaptability complicates treatment, as standard therapies often fail to effectively target metabolically adapted pancreatic cancer cells. Consequently, disrupting the metabolic pathways that facilitate cellular austerity has emerged as a crucial strategy for developing curative pancreatic cancer treatments.

In this context, a detailed phytochemical investigation of *Nelumbo nucifera* petals identified six structurally diverse alkaloids spanning three major chemical classes: benzylisoquinolines, aporphines, and proaporphines. Among these, *N*-nornuciferine (**5**) has garnered particular attention because of its outstanding cytotoxic effects on human pancreatic adenocarcinoma MIA PaCa-2 and KLM-1 cells under nutrient starvation conditions. The exceptionally low PC_50_ values of 0.53 µM and 1.11 µM in the two cell lines highlight its promising efficacy in selectively inducing cell death and morphological changes, specifically in austere environments, suggesting the potential for targeted disruption of the metabolic resilience mechanisms that allow cancer cells to thrive despite nutrient scarcity.

Beyond its preferential cytotoxicity under nutrient deprivation, **5** also inhibits key malignant phenotypes under nutrient-rich conditions at non-cytotoxic concentrations, including cell proliferation, migration, colony formation, and three-dimensional spheroid growth, which collectively support PDAC progression and metastasis. This dual activity profile underscores the potential of this compound as a versatile therapeutic agent capable of impairing pancreatic cancer cell survival in various microenvironmental contexts.

**Figure 11:**
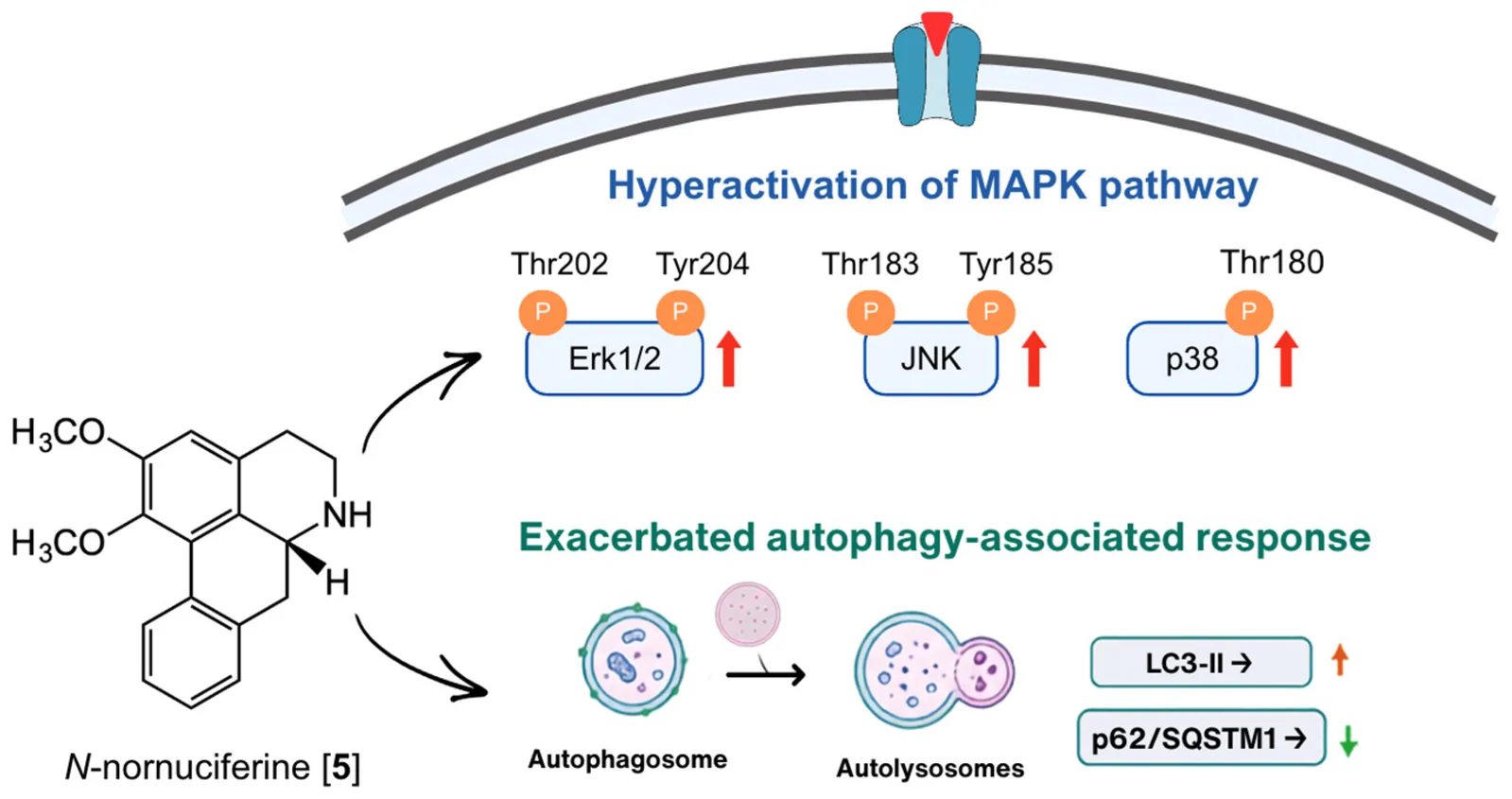
Proposed model of cellular response to *N*-nornuciferine (**5**) under nutrient deprivation. Associated events in the cellular response to *N*-nornuciferine (**5**) include hyperactivation of MAPK family pathways and the triggering of exacerbated autophagic processes, evidenced by LC3-II accumulation and reduced p62/SQSTM1 expression.

Mechanistically, under nutrient deprivation conditions, *N*-nornuciferine (**5**) initiates the phosphorylation of multiple proteins within the MAPK family, indicating the activation of stress-associated MAPK in response to metabolic stress. Furthermore, *N*-nornuciferine (**5**) induced autophagy-related alterations, as evidenced by increased LC3-II levels and decreased p62/SQSTM1 expression levels. The additional accumulation of LC3-II observed with 10 µM *N*-nornuciferine (**5**) in the presence of CQ, compared to CQ alone, further supports the effect of **5** on the autophagy–lysosomal pathway. The proposed cellular responses to **5** treatment under nutrient stress are illustrated in Figure 11.

However, pharmacological inhibition of autophagy using CQ and 3-MA did not significantly mitigate cell viability loss. Thus, the present findings do not establish the mandatory role of autophagy in the cell death process induced by *N*-nornuciferine (**5**). In contrast, *N*-nornuciferine (**5**) did not significantly cleave PARP or caspase-3, and inhibiting caspase activity with Z-VAD-FMK failed to markedly enhance cell viability, suggesting that traditional caspase-dependent apoptosis is unlikely to be a major contributor to the observed cytotoxicity. Overall, **5** induced MAPK phosphorylation and autophagy-associated cellular responses, accompanied by a loss of viability under nutrient deprivation. Thus, *N*-nornuciferine (**5**) may exploit the cellular vulnerabilities related to metabolic stress in pancreatic cancer cells.

Although this study identified an association between MAPK phosphorylation, autophagy, and reduced cell viability, it did not establish a direct causal link between MAPK activation and autophagy. To ascertain whether MAPK activation is essential for the observed effects, specific MAPK pathways must be inhibited through pharmacological and genetic interventions. The analysis of LC3-II/p62, in conjunction with treatments such as CQ and 3-MA, indicates the involvement of the autophagy–lysosomal pathway; however, these methods do not directly evaluate autophagic flux. Further validation could be achieved using alternative techniques, such as tandem fluorescent LC3 reporters. Moreover, apoptotic markers were assessed only at a single 6 h time point; a time-course analysis (e.g., 3, 6, 12, and 24 h) would help determine whether transient or delayed apoptotic activation occurs, representing an important direction for future research.

The absence of PARP and caspase-3 cleavage, together with the ineffectiveness of Z-VAD-FMK in restoring cell viability, indicates that classical caspase-dependent apoptosis is unlikely to be a major contributor to *N*-nornuciferine (**5**)-induced cytotoxicity, although other forms of regulated cell death cannot be fully excluded based on the current dataset. Consistent with this, Annexin V/PI staining performed for 6 h under nutrient deprivation did not reveal detectable Annexin V- or PI-positive populations in either control or *N*-nornuciferine (**5**)-treated cells, while heat-shocked cells confirmed the assay’s responsiveness as a positive control (See Supplementary Materials, PDF S1, Figure S23). Ferroptosis and necroptosis were not directly investigated in the present study, and dedicated markers (e.g., lipid peroxidation, p-MLKL/p-RIPK3) are required to formally exclude these pathways as contributors to the observed loss of membrane integrity. Therefore, further research is required to fully elucidate the specific interactions between MAPK signaling, autophagy, and *N*-nornuciferine-induced cell death.

The current study identified *N*-nornuciferine (**5**) as a promising lead compound for the development of next-generation drugs, leveraging its anti-austere properties to address the metabolic vulnerabilities of pancreatic cancer. Its capacity to selectively target cancer cells under nutrient stress while inhibiting other significant malignant phenotypes in nutrient-rich conditions underscores its potential to address the complex challenges of pancreatic tumor heterogeneity and metabolic plasticity. Future research should focus on elucidating the detailed molecular mechanisms of *N*-nornuciferine, optimizing its pharmacological properties, and evaluating its efficacy in preclinical models. These steps are crucial for advancing this compound towards clinical application as a therapeutic agent against pancreatic cancer.

## 4. Materials and Methods

### 4.1. General Experimental Procedures and Reagents

Medium-pressure liquid chromatography (MPLC) was performed using a Büchi MPLC C-605 dual-gradient pump system with normal-phase silica gel (Silica Gel 60N; Kanto Chemical, Tokyo, Japan). Analytical and preparative TLC were performed on pre-coated silica gel 60F254 and RP-18F254 plates (0.25 or 0.50 mm, Merck KGaA, Darmstadt, Germany) and pre-coated silica gel 70 PF254 plates (0.75 mm, FUJIFILM Wako, Ltd., Osaka, Japan). Infrared (IR) spectra were obtained using a JASCO FT/IR-460 Plus spectrophotometer (JASCO Corporation, Tokyo, Japan). One-dimensional NMR spectra were recorded on a JEOL ECA400 Delta spectrometer (JEOL Ltd., Tokyo, Japan) using chloroform-d and methanol-d_4_ as solvents, and the chemical shifts were expressed as *δ* values.

### 4.2. Chemicals and Antibodies

All reagents were procured from commercial suppliers and used without further purification, unless otherwise noted. The following materials were used for cell culture: Dulbecco’s modified Eagle medium (DMEM; FUJIFILM Wako, Ltd., Osaka, Japan; Cat. No. #041-29775), DMEM/Ham’s F-12 (FUJIFILM Wako, Osaka, Japan; Cat. No. #048-29785), RPMI-1640 (NACALAI TESQUE, INC., Kyoto, Japan; Cat. No. #30264-85), fetal bovine serum (FBS; Gibco, Thermo Fisher Scientific, Tokyo, Japan; Cat. No. #10270-106), antibiotic-antimycotic solution (Sigma-Aldrich, Tokyo, Japan; Cat. #A5955), and phosphate-buffered saline (PBS; FUJIFILM Wako, Osaka, Japan; Cat. #166-23555). Nutrient-deprived medium (NDM) was prepared using glucose-free DMEM (FUJIFILM Wako, Osaka, Japan; Cat. #042-32255) containing a 1% antibiotic-antimycotic solution without FBS. All test alkaloids were dissolved in dimethyl sulfoxide (DMSO; Sigma-Aldrich, Tokyo, Japan; Cat. #D2650) to prepare a 10 mM stock solution. Chloroquine (CQ; FUJIFILM Wako, Osaka, Japan; Cat. #038-17971) was dissolved in distilled water for storage. 3-Methyladenine (3-MA; Selleck Biotech Co., Ltd., Kanagawa, Japan; Cat. #S2767) was dissolved in DMSO to prepare a stock solution. The pan-caspase inhibitor Z-VAD-FMK (Selleck Chemicals LLC, Cat. #S7023) was prepared in DMSO as a stock solution. Primary antibodies were purchased from Cell Signaling Technology, Inc. (Danvers, MA, USA) and included the MAPK family rabbit antibody sampler kit (#9926), consisting of p44/42 MAPK (Erk1/2, #4695), SAPK/JNK (#9252), and p38 MAPK (#8690); and the phospho-MAPK family antibody sampler kit (#9910), consisting of phospho-p44/42 MAPK (Erk1/2, Thr202/Tyr204, #4370), phospho-SAPK/JNK (Thr183/Tyr185, #4668), phospho-p38 MAPK (Thr180/Tyr182, #4511); LC3 (#2775), PARP (46D11) rabbit mAb (#9532; 116 and 89 kDa), Caspase-3 (D3R6Y) rabbit mAb (#14220; 35, 19, and 17 kDa), and β-tubulin (#2146). Primary antibody p62/SQSTM1 rabbit mAb (Cat. #NBP1-48320SS; 56 kDa) was purchased from Novus Biologicals (Bio-Techne, Tokyo, Japan). Horseradish peroxidase (HRP)-conjugated anti-rabbit IgG (Dako, Agilent Technologies, Glostrup, Denmark; Cat. #P0448) was used as the secondary antibody for immunoblotting.

### 4.3. Plant Material, Extraction, and Isolation

*Nelumbo nucifera* petals were collected in May 2019 from Bang Decha, Prachinburi, Thailand. The plant was taxonomically identified by Mrs. Benjawan Leenin, Chief of the Traditional Knowledge Center, Chao Phraya Abhaibhubejhr Hospital Foundation. A voucher specimen (ABH15) was deposited in the herbarium at the same institution.

Dried petals (3 kg) were extracted with 95% ethanol, yielding 300 g of crude extract, as described in our previous publication [13]. Extraction, partitioning, and initial fractionation steps were concurrently performed in these studies. However, several compounds isolated during this period were not included in the earlier report and were characterized and discussed in this study. Briefly, the ethanol extract was suspended in water (H_2_O) and partitioned with EtOAc, resulting in EtOAc-soluble (180 g) and H_2_O-soluble (120 g) fractions, respectively. The H_2_O-soluble fraction was acidified with 1% aqueous acetic acid and extracted with dichloromethane (CH_2_Cl_2_). The CH_2_Cl_2_-soluble portion was then basified using 10% ammonium hydroxide (NH_4_OH) and re-extracted with CH_2_Cl_2_ to obtain a crude alkaloid fraction (CAF, 3 g).

CAF was subjected to silica gel column chromatography using a CH_2_Cl_2_/methanol (MeOH) gradient mixture (0–100%), yielding seven major fractions (Fr. 1, 109 mg; Fr. 2, 900 mg; Fr. 3, 376 mg; Fr. 4, 711 mg; Fr. 5, 148 mg; Fr. 6, 55 mg). Fraction 1 (109 mg) was further chromatographed on silica gel by MPLC using a CH_2_Cl_2_/1% NH_4_OH in MeOH gradient mixture (0–100%) to yield five subfractions (Fr. 1–1, 9 mg; Fr. 1–2, 50.9 mg; Fr. 1–3, 4.1 mg; Fr. 1–4, 12.3 mg; Fr. 1–5, 36.4 mg). Subfraction 1–2 (50.9 mg) was identified as (–)-nuciferine (**4**, 15.9 mg). Fraction 3 (376 mg) was separated on silica gel by MPLC using CH_2_Cl_2_/1% NH_4_OH in a MeOH gradient system (0–100%) to obtain five subfractions (Fr. 3–1, 82 mg; Fr. 3–2, 73 mg; Fr. 3–3, 102 mg; Fr. 3–4, 63 mg; Fr. 3–5, 35 mg). Subfraction 3–1 (82 mg) was re-chromatographed on silica gel by MPLC using CH_2_Cl_2_/1% NH_4_OH in a MeOH gradient system (0–100%) to obtain (+)-pronuciferine (**6**, 10.9 mg). Subfraction 3–2 (73 mg) was purified by preparative TLC using 1% NH_4_OH in Acetone/CH_2_Cl_2_ (33:67) to isolate (−)-*N*-nornuciferine (**5**, 10.6 mg). Subfraction 3–3 (102 mg) was purified by preparative TLC with 1% NH_4_OH in Acetone/CH_2_Cl_2_ (20:80, then 2:78) to yield (−)-4′-*O*-methylcoclaurine (**2**, 10.9 mg). Subfraction 3–4 (63 mg) was purified by preparative TLC with 1% NH_4_OH in Acetone/CH_2_Cl_2_ (22:78, then 25:75), resulting in (−)-norarmepavine (**1**, 20.3 mg). Subfraction 3–5 (35 mg) was purified by preparative TLC with 1% NH_4_OH in MeOH/CH_2_Cl_2_ (7:93), yielding (−)-isoliensinine (**3**, 10.2 mg). All known compounds were identified by comparing their physical data (^1^H NMR, ^13^C NMR, and [α]_D_ values). We measured the optical rotation ([α]_D_) for all isolated compounds and report it alongside the corresponding NMR data in the Supplementary Material to support unambiguous structural and stereochemical assignment.

### 4.4. Cell Culture

The human pancreatic cancer cell lines MIA PaCa-2 (RBRC-RCB2094) and KLM-1 (RBRC-RCB2138) were obtained from the RIKEN BRC Cell Bank in Tsukuba, Japan. The immortalized mouse colonic epithelial cells (MCE301) were established and characterized by Professor Yoshiaki Tabuchi, Division of Molecular Genetics Research, Life Science Research Center, Organization for Promotion of Research, University of Toyama. These cells were maintained in DMEM for MIA PaCa-2, RPMI-1640 for KLM-1, and DMEM/ Ham’s F12 for MCE301 cells, with the addition of 10% FBS and 1% antibiotic-antimycotic solution, at a temperature of 37 °C within a humidified incubator with 5% CO_2_. To maintain uniformity, the cell passages were preserved at −80 °C.

### 4.5. Preferential Cytotoxicity Assay

The cytotoxic effects of the isolated compounds were evaluated on MIA PaCa-2, KLM-1, and immortalized colonic epithelial MCE301 cells, following a previously established protocol. Specifically, the cell concentration was maintained at 2 × 10^5^ cells/mL, with 100 µL aliquots dispensed into each well of 96-well plates (Corning, Tewksbury, MA, USA; Cat. #3596) and incubated for 24 h thereafter. Post-incubation, the cells were washed with PBS, and the medium was replaced with either NDM or nutrient-rich medium (DMEM for MIA PaCa-2 cells, RPMI-1640 for KLM-1 cells) containing the test compounds at serial dilutions (0–100 µM; maintaining a final DMSO concentration of 0.1%), and DMEM/Ham’s F-12 medium with serially diluted test compounds for MCE301. The cells were subsequently incubated at 37 °C in a 5% CO_2_ atmosphere for an additional 24 h. Thereafter, the medium was replaced with 100 µL growth medium according to cell lines containing 10% Cell Counting Kit-8 solution (CCK-8, Cat. #CK04, Dojindo, Kumamoto, Japan). Following a 3-h incubation, absorbance was measured at 450 nm using a microplate reader (Bio-Rad Laboratories K.K., Tokyo, Japan; Model 680). The formula employed to calculate cell viability was as follows:

Cell viability [%] = [Abs_(test sample)_ − Abs_(blank)_]/[Abs_(control)_ − Abs_(blank)_] × 100

Abs_(blank)_ refers to the absorbance measured in wells containing only the medium, serving as a baseline control. Each experiment was repeated three times. The PC_50_ values indicate the concentration required to cause 50% cell death under NDM conditions without toxicity in nutrient-rich conditions. In contrast, the IC_50_ values denote the concentration of the compound needed to decrease cancer cell viability by 50% in normal nutrient medium. These values were calculated using GraphPad Prism software (version 10). In subsequent experiments, compound **5** was tested at concentrations ranging from 0.5 to 10 µM in NDM to explore its anti-austerity effect. To assess the anti-proliferative, anti-metastatic, colony formation inhibitory, and spheroid formation inhibitory effects in DMEM, higher non-cytotoxic concentrations below the IC_50_ value, between 1.25 and 50 µM, were used. This approach was adopted to ensure assay specificity and prevent confounding effects associated with general cytotoxicity.

### 4.6. Morphological Analysis

MIA PaCa-2 cells (2 × 10^5^ in 2 mL medium) were seeded into 35 mm dishes and left to adhere overnight. Subsequently, the cells were exposed to compound **5** at concentrations of 0.5 and 1.0 µM in NDM at 37 °C for 24 h. Concurrently, live-cell imaging was conducted using the CytoSMART Lux2 (Axion BioSystems, Eindhoven, The Netherlands) at 15-min intervals for 24 h, with specific snapshots being analyzed (Supplementary Movie S1). After the 24-h treatment, the cells were stained with ethidium bromide/acridine orange (EB/AO, Sigma-Aldrich; Cat. #E2889 and #A6014 (mixed in a 1:1 ratio, 100 µg/mL each) for 15 min and visualized using an EVOS FL Auto Imaging System (Thermo Fisher Scientific, Waltham, MA, USA) equipped with GFP and RFP filters. Representative fields were selected based on consistency with the overall phenotype observed across replicate wells rather than the magnitude of the treatment effect, and quantification was not performed in a blinded manner. However, identical acquisition and processing settings were uniformly applied across all treatment groups.

### 4.7. Cell Proliferation

MIA PaCa-2 cells, at a density of 3000 cells per 100 µL per well, were placed in a 96-well plate and maintained at 37 °C with 5% CO_2_ for 24 h. Subsequently, the cells were treated with compound **5** at concentrations ranging from 0 to 50 µM, with a final DMSO concentration of 0.1% in DMEM, for 72 h. The confluence percentage was evaluated through algorithmic analysis using the OMNI device (Axion BioSystems, Eindhoven, The Netherlands), which captured bright-field images every 3 h (Supplementary Movie S2). All experiments were performed in triplicate.

### 4.8. Cell Migration

To assess cell migration, a 2-well culture insert assay (Ibidi, Nippon Genetics Co., Ltd., Tokyo, Japan; Cat. #80209) was used following standard procedures. MIA PaCa-2 cells, at a concentration of 1 × 10^6^ cells/mL in DMEM, were added to 2-well culture inserts placed in 35-mm dishes, with 70 µL allocated per well. The cells were then incubated for 24 h at 37 °C in an atmosphere containing 5% CO_2_. After incubation, the inserts were removed to form an approximately 500-µm gap devoid of cells. The cells were rinsed with PBS, and the medium was replaced with either DMEM alone (as a control) or DMEM with **5** (25 and 50 µM). Time-lapse imaging was conducted using the CytoSMART Lux2 (version 33.15) at 15-min intervals over a 48-h duration within a 5% CO_2_ incubator (Supplementary Movie S3). The open area, calculated as a percentage of the initial gap, was measured from bright-field images using CytoSMART Lux2 software.

### 4.9. Colony Formation

To conduct the colony formation assay, 24-well plates (Corning, Tewksbury, MA, USA; Cat. #353047) were used. MIA PaCa-2 cells were initially seeded at a concentration of 2.5 × 10^3^ cells per well in 1 mL of DMEM and maintained at 37 °C with 5% CO_2_ for 24 h. After this period, the cells were exposed to different concentrations (0, 1.25, 2.5, 5, 10, and 20 µM) of compound **5** in DMEM for 24 h. Post-treatment, the medium was exchanged for fresh DMEM, and the cells were left to grow for 10 d. The colonies were fixed using 4% formaldehyde (FUJIFILM Wako, Osaka, Japan; Cat. #064-00406) and stained with 0.5% crystal violet (Sigma-Aldrich; Cat. #C0775) for 15 min, followed by rinsing with water several times. The colony area percentage was measured using the ImageJ “ColonyArea” plugin. Each experiment was conducted four times.

### 4.10. Western Blot Analysis

MIA PaCa-2 cells were initially plated at a concentration of 5 × 10^5^ cells/mL in 2 mL of DMEM per well within 6-well plates (Corning, Cat. #3516) and incubated overnight. On the subsequent day, the cells were treated with **5** in either NDM (0, 2.5, 5, and 10 µM) or DMEM (0 and 10 µM). For the combination treatment, CQ (50 µM) or 3-MA (3 mM) was introduced alongside **5** (0, 5, and 10 µM) exclusively under NDM conditions. The incubation was continued for 6 h at 37 °C with 5% CO_2_. After incubation, the cells were rinsed with ice-cold PBS and lysed using RIPA buffer (Thermo Fisher Scientific, Cat. #89900), which was enhanced with a protease inhibitor cocktail (Sigma-Aldrich; Cat. #P8340), 1 mM sodium orthovanadate, 40 mM β-glycerophosphate, and 1 mM phenylmethanesulfonyl fluoride. The lysates were maintained on ice for 30 min before centrifugation at 13,000 rpm for 12 min at 4 °C. The obtained supernatants were stored at −20 °C for later analysis.

Protein levels were measured using the BCA assay (Thermo Fisher Scientific, Cat. #23225). Proteins (20 µg per lane) were separated by SDS-PAGE on 8% or 15% polyacrylamide gels and then transferred to PVDF membranes (Bio-Rad, Cat. #1620177). The membranes were blocked with 5% skim milk in Tris-buffered saline with 0.1% Tween 20 (TBST, Sigma-Aldrich; Cat. #P9416) for 1 h, followed by overnight incubation at 4 °C with primary antibodies. After three washes with TBST, the membranes were treated with horseradish peroxidase (HRP)-conjugated secondary antibodies for 1 h at room temperature. Protein bands were detected using enhanced chemiluminescence (ECL) solution (GE Healthcare, Chicago, IL, USA; Cat. #RPN2232) and captured using an ImageQuant LAS 4000 system (GE Healthcare). Band intensities were quantified using the ImageJ software and normalized against β-tubulin levels.

### 4.11. Pharmacological Inhibition of Autophagy and Caspase-Dependent Apoptosis

Mia PaCa-2 cells were placed in a 96-well plate to conduct a preferential assay. To block autophagy, the cells underwent pretreatment with either 50 μM chloroquine (CQ) or 3 mM 3-methyladenine (3-MA) for 1 h. *N*-nornuciferine (**5**) was then administered at various concentrations (0, 2.5, 5, and 10 μM) for 6 h in a nutrient-deprived medium (NDM). To inhibit caspase-dependent apoptosis, cells were pretreated with the pan-caspase inhibitor Z-VAD-FMK (20 μM) for 1 h before exposure to *N*-nornuciferine (**5**) for 6 h. Cells treated with vehicle served as controls. After treatment, cell viability was evaluated using the CCK-8 assay, according to the manufacturer’s guidelines. Absorbance was recorded at 450 nm, and cell viability was calculated as a percentage of that of the untreated control.

### 4.12. Spheroid Formation Assay

MIA PaCa-2 cells were seeded at a density of 1000 cells/well in a 96-well ultralow-attachment PrimeSurface plate (Fujifilm Wako, Osaka, Japan; Cat. No. MS-9096M). To promote cell aggregation and spheroid formation, the plate was centrifuged in a swinging-bucket rotor at 1500 rpm for 10 min at room temperature. Spheroid growth was monitored every 6 h using an OMNI device. On day 3, the spheroids were treated with **5** at concentrations of 5, 10, and 20 µM or with vehicle control (Supplementary Movie S4). On day 6, half of the culture medium was replaced with fresh DMEM, and the spheroids were cultured until day 10. No statistically significant differences in the spheroid projected area or estimated volume were observed among the experimental groups at baseline (day 3, before treatment initiation; *p* > 0.05, two-way ANOVA), confirming a uniform starting spheroid size across all conditions (see Supplementary Materials for quantification of spheroid volume).

At the end of the experiment, the spheroids were stained with ethidium bromide (EB) and acridine orange (AO). Fluorescence images were acquired using a fluorescence microscope. EB/AO staining was used as a qualitative endpoint assessment of membrane integrity and cell viability; fluorescence intensity and red/green signal ratios were not quantified.

Original bright-field images were exported for quantitative analyses. The spheroid projected area was measured using the “Spheroid Area” macro in ImageJ software. The macro was applied in batch mode to all images using identical, predefined analysis parameters. The equivalent spheroid diameter was calculated from projected area (A) according to $d=2\sqrt{A/\pi }$. Estimated spheroid volume (V) was calculated by assuming spherical geometry. The equivalent radius (r) was calculated as $r=\sqrt{A/\pi }$, and volume was calculated according to $V=\frac{4}{3}\pi r^{3}$. When *A* was expressed in $\mu m^{2}$, estimated volume in ${mm}^{3}$ was calculated as:

$$V=\frac{4A^{3/2}}{3\sqrt{\pi }}\times 10^{-9}.$$

Because the volume was inferred from two-dimensional bright-field images, it represents an estimated rather than a directly measured three-dimensional parameter.

### 4.13. Statistical Analysis

GraphPad Prism (version 10; GraphPad Software, San Diego, CA, USA) was utilized for conducting statistical analyses. To assess preferential cytotoxicity and colony formation, one-way analysis of variance (ANOVA) was employed, followed by Dunnett’s multiple comparison test. Differences in estimated spheroid volume and cell proliferation confluence percentage were evaluated using a two-way ANOVA with Dunnett’s multiple comparison test. For Western blot data, either a one-way ANOVA or an unpaired *t*-test was applied (*n* = 3). A *p*-value of less than 0.05 was considered statistically significant (* *p* < 0.05, ** *p* < 0.01, and *** *p* < 0.001).

## 5. Conclusions

*N*-nornuciferine (**5**), an aporphine alkaloid derived from *Nelumbo nucifera*, has shown promising anti-austerity activity against MIA PaCa-2 and KLM-1 human pancreatic cancer cell lines. Under nutrient-deprived conditions, **5** exhibited preferential cytotoxicity against these cell lines, with PC_50_ values of 0.53 and 1.11 µM, respectively. Treatment with **5** induced cell death and distinct morphological changes in nutrient-stress environments, while significantly inhibiting MIA PaCa-2 cell proliferation, migration, colony formation, and spheroid growth in nutrient-rich conditions. Mechanistic studies demonstrated increased phosphorylation of multiple MAPK family proteins, together with autophagy-associated changes, including LC3-II accumulation and p62/SQSTM1 reduction, under nutrient deprivation. However, pharmacological inhibition of autophagy with CQ or 3-MA did not restore **5**-induced cytotoxicity, and Z-VAD-FMK also failed to rescue cell viability. In addition to the lack of PARP or caspase-3 cleavage and Annexin V/PI-positive staining, these findings suggest that conventional caspase-dependent apoptosis is unlikely to be a major contributor to the cytotoxic response. The ability of **5** to induce MAPK activation and autophagy-associated cellular responses and selectively target pancreatic cancer cells under metabolic stress underscores its therapeutic potential for overcoming resistance to apoptosis-refractory pancreatic ductal adenocarcinoma.

## Figures and Tables

**Table 1:** Preferential cytotoxicity (PC_50_) of alkaloids isolated from *Nelumbo nucifera* petals against MIA PaCa-2 cells under nutrient-deprived conditions and their cytotoxicity (IC_50_) under DMEM conditions after 24 h.

Compound	NDM (PC_50_ µM)	DMEM (IC_50_ µM)	Selectivity *^c^*
**1**	40.0	>100	nd
**2**	49.1	>100	nd
**3**	3.50	>100	nd
**4**	0.11	31.9	290
**5**	0.53	130	245.3
**6**	0.95	98.1	103.3
arctigenin *^a^*	0.10	>100	>1000
gemcitabine *^b^*	>100	>100	nd

*^a^* positive control, *^b^* standard reference, *^c^* selectivity: IC_50_ value for Dulbecco’s Modified Eagle Medium (DMEM)/PC_50_ value for nutrient-deprived medium (NDM), nd: not determined.

## Data Availability

The original contributions presented in this study are included in the article/Supplementary Materials. Further inquiries can be directed to the corresponding author.

## Supplementary Materials

The following supporting information can be downloaded at: https://doi.org/10.5281/zenodo.22741550. **PDF S1:** ^1^H, ^13^C NMR spectra, and optical rotation data of the six isolated compounds, time-lapse imaging of MIA PaCa-2 live cells, cell proliferation, cell migration, and spheroid formation assays. **PDF S2:** Uncropped Western blot image experiments. **Movie S1:** Time-lapse live-cell imaging of MIA PaCa-2 cells treated with *N*-nornuciferine (**5**) (24 h, 15-min intervals). **Movie S2:** Time-lapse imaging of MIA PaCa-2 cell proliferation treated with *N*-nornuciferine (**5**) (72 h, 3-h intervals). **Movie S3:** Time-lapse imaging of MIA PaCa-2 cell migration treated with *N*-nornuciferine (**5**) (48 h, 15-min intervals). **Movie S4:** Time-lapse imaging of MIA PaCa-2 spheroid growth inhibition by *N*-nornuciferine (**5**) (10 d, 6-h intervals).
